# MM quadruply bonded complexes supported by vinylbenzoate ligands: synthesis, characterization, photophysical properties and application as synthons†
†Electronic supplementary information (ESI) available: Transient absorption plots and kinetics, time resolved IR plots, NIR emission. CCDC 1015787 and 1015788. For ESI and crystallographic data in CIF or other electronic format see DOI: 10.1039/c4sc02542c



**DOI:** 10.1039/c4sc02542c

**Published:** 2015-01-13

**Authors:** Samantha E. Brown-Xu, Malcolm H. Chisholm, Christopher B. Durr, Thomas F. Spilker, Philip J. Young

**Affiliations:** a The Ohio State University , Department of Chemistry and Biochemistry , 100 W. 18th Avenue , Columbus , Ohio 43202 , USA . Email: Chisholm@chemistry.ohio-state.edu ; Tel: +1-614-292-7216

## Abstract

From the reactions between M_2_(T^i^PB)_4_ compounds and *meta* and *para*-vinylbenzoic acids (2 equiv.) in toluene at room temperature the compounds *trans*-M_2_(T^i^PB)_2_L_2_, where L = *m*-vinylbenzoate **1A** (M = Mo) and **1B** (M = W) and T^i^PB = 2,4,6-triisopropylbenzoate, and where L = *p*-vinylbenzoate **2A** (M = Mo) and **2B** (M = W) have been isolated. Compounds **1A** and **2A** have been shown to undergo Heck carbon–carbon coupling reactions with phenyliodide to produce *trans*-Mo_2_(T^i^PB)_2_(O_2_CC_6_H_4_-*m*-CH=CH–C_6_H_5_)_2_, **3A** and *trans*-Mo_2_(T^i^PB)_2_(O_2_CC_6_H_4_-*p*-CH=CH–C_6_H_5_)_2_, **4A**. The molybdenum compounds **1A** and **2A** have been structurally characterized by single crystal X-ray crystallography. All the new compounds have been characterized by ^1^H NMR, IR, UV-visible absorption and emission spectroscopy, high resolution MALDI-TOF MS, fs- and ns-transient absorption spectroscopy and fs-time-resolved IR spectroscopy. Electronic structure calculations employing density functional theory, DFT, and time-dependent DFT have been employed to aid in the interpretation of spectral data. All compounds show intense absorptions in the visible region corresponding to M_2_δ to Lπ* charge transfer transitions. The lifetimes of the ^1^MLCT state fall in the range of 1–10 ps and for the molybdenum complexes the T_1_ states are ^3^δδ* with lifetimes ∼50 μs while for the tungsten complexes the T_1_ are ^3^MLCT with lifetimes in the range of 3–10 ns.

## Abstract 2

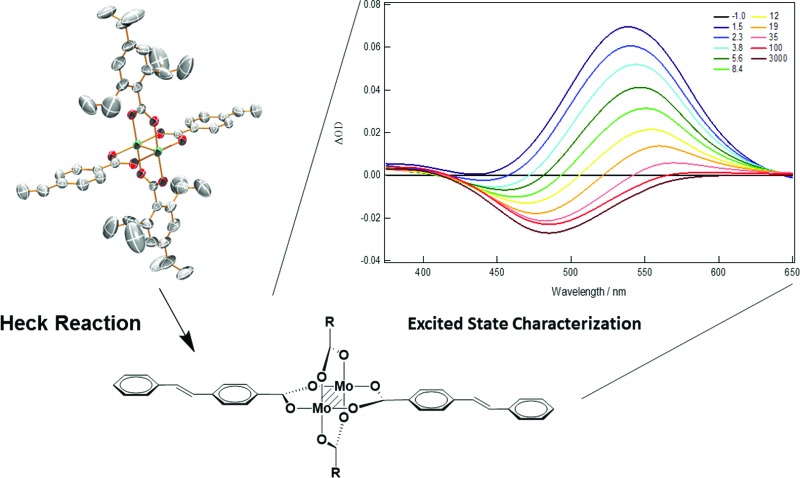
MM complexes as potential synthons for the development of higher order extended structures *via* Heck coupling reactions, exhibiting interesting photophysical properties.

## Introduction

The field of organic electronics based on conducting organic polymers is a rapidly developing field embracing both chemistry and materials science. Poly-*p*-phenylene vinylene (PPV) is one of the most studied conducting organic polymers to date and has been at the center of the development of organic electronics.^1–7^ Friend and coworkers discovered the polymer light emitting diode with PPV showing yellow-green electroluminescence in 1990.^1^ The first bulk heterojunction photovoltaic cell was made with PPV derivatives as the light absorbing layer by Heeger^3^ and Friend^8^ in 1995. The incorporation of transition metals into PPV derivatives offers the opportunity to couple the properties of the transition metal with those of the polymer and was first achieved with ruthenium and osmium *via* the Heck reaction and the properties of these complexes have been explored in detail.^9^ They possess the π–π* transition of the parent polymer and also an MLCT transition from the metal to the bipyridine that it is bound to in the polymer. The luminescence of these systems is dominated by the ^3^MLCT state traditionally present in metal–pyridyl systems.^10^ Ruthenium and osmium PPV derivatives were also examined as light emitting layers in polymer light emitting diodes (PLEDs) and it was shown the emission was largely MLCT based. The metal can also be used to toggle between conjugated and nonconjugated organic polymers, where coordination of the metal forces the planarization of the polymer and enhances the conjugation.^11^

With this in mind, it seems appropriate to explore the incorporation of MM quadruply bonded complexes into PPV derivatives by way of carbon–carbon cross coupling reactions, such as the Heck reaction. The use of carbon–carbon cross coupling reactions on the periphery of transition metal complexes has been previously explored;^12^ however, there is a limited scope of these reactions on MM paddlewheel complexes. Ren and company have shown the Heck reaction can be successfully employed on Ru_2_ formamidinate compounds to add a styrene functionality. Reactions on the quadruply bonded Mo_2_ tetracarboxylate complexes have proven more difficult due to the kinetic lability of the carboxylate ligands. The initial example of this has shown that the Sonogashira reaction can be employed on the periphery of MM compounds successfully without compromising the quadruply bonded core.^13^

We have previously shown that the attachment of π-conjugated organic units such as oligothiophenes *via* carboxylate units to the MM quadruply bonded centers have intense ^1^MLCT transitions that may be tuned to span the entire visible region of the spectrum.^14^ Moreover, the ^1^MLCT states have relatively long lifetimes, typically in the range of 1–20 ps. In this paper, a series of Mo_2_ and W_2_ bis–bis complexes supported by vinylbenzoate ligands have been prepared and their ground state and excited state photophysical properties are explored. It is also shown that the Mo_2_ complexes are viable synthons for the preparation of higher order extended structures that possess very interesting photophysical properties.

## Results and discussion

### Synthesis

**Scheme 1:**
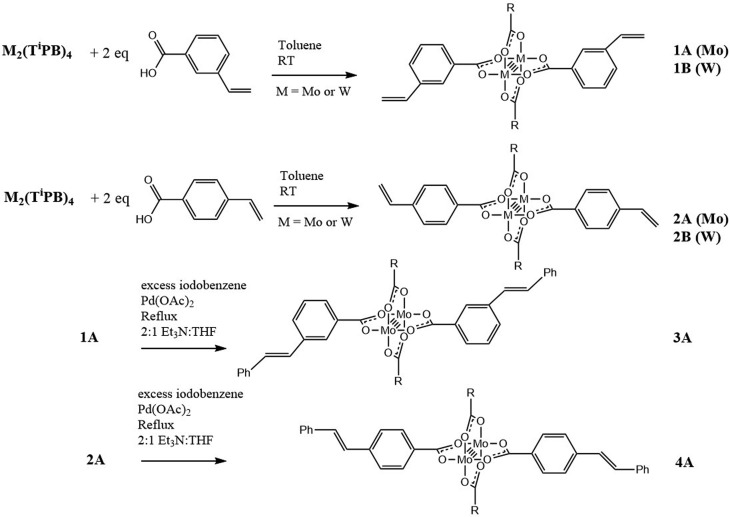
Synthesis of new vinylbenzoate supported M_2_ complexes, where M = Mo (**A**) or W (**B**), and R = 2,4,6-triisopropylphenyl.

The synthesis of the new compounds is outlined in Scheme 1. The *trans*-M_2_ compounds **1A–2B** were prepared by the ligand exchange reaction between M_2_T^i^PB_4_ with the respective carboxylic acid, either 3-vinylbenzoic acid (**1**) or 4-vinylbenzoic acid (**2**), where M = Mo (**A**) or W (**B**) and T^i^PB = 2,4,6-triisopropylbenzoate. The reactions were performed in toluene, where the desired product was slightly soluble. The desired product was then isolated by reducing the volume of the solvent *in vacuo* and precipitation by the addition of hexanes. They are highly colored solids that are yellow (**1A**), orange (**2A**), maroon (**1B**) and blue (**2B**). The new compounds are air sensitive and soluble in toluene, THF, and DCM. They gave molecular ions in the MALDI-TOF MS and ^1^H NMR data are consistent with their formulations. Further characterization details are given in the Experimental. The new compounds **1A** and **2A** were then subjected to further reactions. A Heck cross coupling reaction was successfully performed on both Mo_2_ complexes with excess iodobenzene in the presence of palladium(ii) acetate, Et_3_N and heat. For **1A**, there was no visible color change; however, for **2A**, the solution went from orange to red. The products were isolated by filtration of the reaction mixture through a bed of celite. The solvent was then reduced to a minimal amount and hexanes were added to precipitate the product. The desired products were purified further by recrystallization. **3A** and **4A** gave molecular ions in the MALDI-TOF MS and ^1^H NMR consistent with their formulations. Further characterization details are given in the Experimental. MALDI-TOF MS and ^1^H NMR plots can be seen in the ESI.†

Reactions were attempted on the W_2_ compounds; however, they are more sensitive to oxidation than their Mo_2_ counterparts and the W_2_ desired coupled products were not obtained. Further studies pertaining to C–C coupling reactions involving W_2_-quadruply bonded complexes are underway.

### Single crystal X-ray structures

**Fig. 1:**
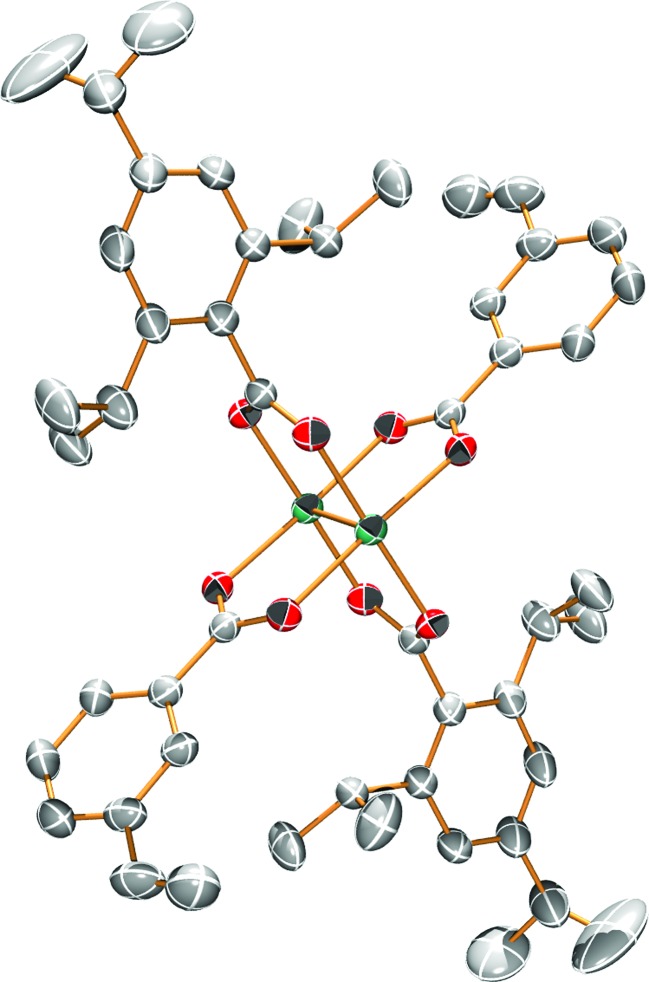
ORTEP structure of Mo_2_T^i^PB_2_(3-vinylbenzoate)_2_ [**1A**]. Hydrogens and solvent omitted for clarity. Thermal ellipsoids shown at 50%.

**Fig. 2:**
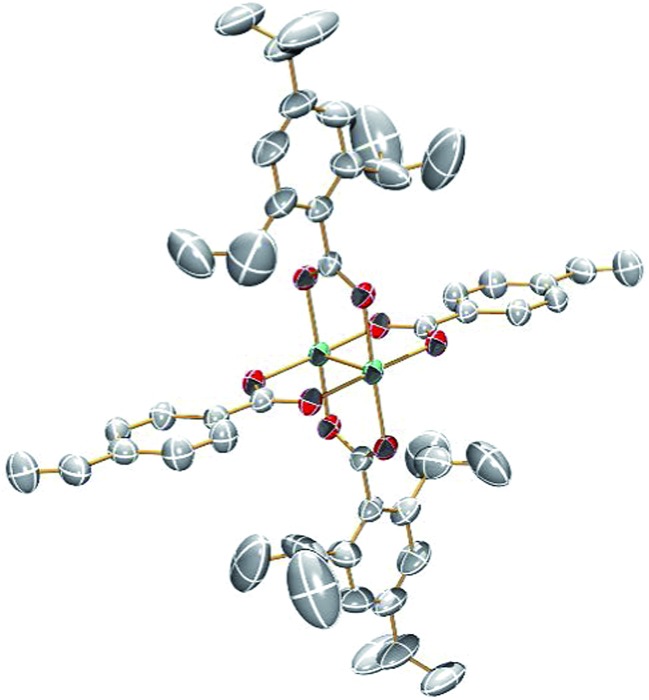
ORTEP structure of Mo_2_T^i^PB_2_(4-vinylbenzoate)_2_ [**2A**]. Hydrogens, disorder and solvent omitted for clarity. Thermal ellipsoids shown at 50%.

Single crystals suitable for X-ray analysis for **1A** and **2A** were grown from vapor diffusion of hexanes into a concentrated THF solution. The molecular structures of **1A** and **2A** are shown in Fig. 1 and 2, respectively. Both structures have a center of inversion and the T^i^PB units are twisted such that the phenyl rings are out of conjugation with the –CO_2_ units with dihedral angles of 79.6° and 83.3°, respectively. The vinylphenyl unit, however, is coplanar with its –CO_2_ unit making it in conjugation with the Mo_2_ center with dihedral angles of 11.3° and <1° in **1A** and **2A**, respectively. The angle between the vinyl and phenyl units in **1A** is 4.5° and in **2A** is <1°. The Mo–Mo and Mo–O bond lengths are ∼2.1 Å and the Mo_2_(O_2_CR)_4_ core is typical for Mo_2_^4+^ complexes.^15^ For **2A**, the terminal vinyl carbon shows disorder in two positions, but Fig. 2 shows only one position. The axial sites of the MoMo unit are occupied by coordinating THF with a THF–O···Mo distance of ∼2.6 Å. A table of select crystallographic information can be found in the ESI.†

### Electronic structure calculations

In order to assist in the interpretation of the spectral data, electronic structure calculations have been performed on model complexes of the form *trans*-M_2_(O_2_CH)_2_(vinylbenzoate)_2_, where T^i^PB is substituted for formate. Density functional theory (DFT) and time-dependent DFT (TD-DFT) have been employed to this end. The TD-DFT calculations predict these compounds to possess several intense, fully-allowed transitions. There is a metal-to-ligand charge transfer (MLCT) from the M_2_δ to vinylbenzoate π*, a MLCT from the M_2_δ to the formate π* and a π–π* transition based on the vinylbenzoate ligands.

Calculations were performed on model complexes **1A′**, **1B′**, **2A′**, **2B′**, **3A′** and **4A′** where the T^i^PB moiety has been replaced by a formate to save on computational resources. This is a reasonable assumption because the phenyl ring of the T^i^PB is twisted ∼90° out of conjugation with respect to the M_2_ center which effectively removes its contribution to the electronic structure.

**Fig. 3:**
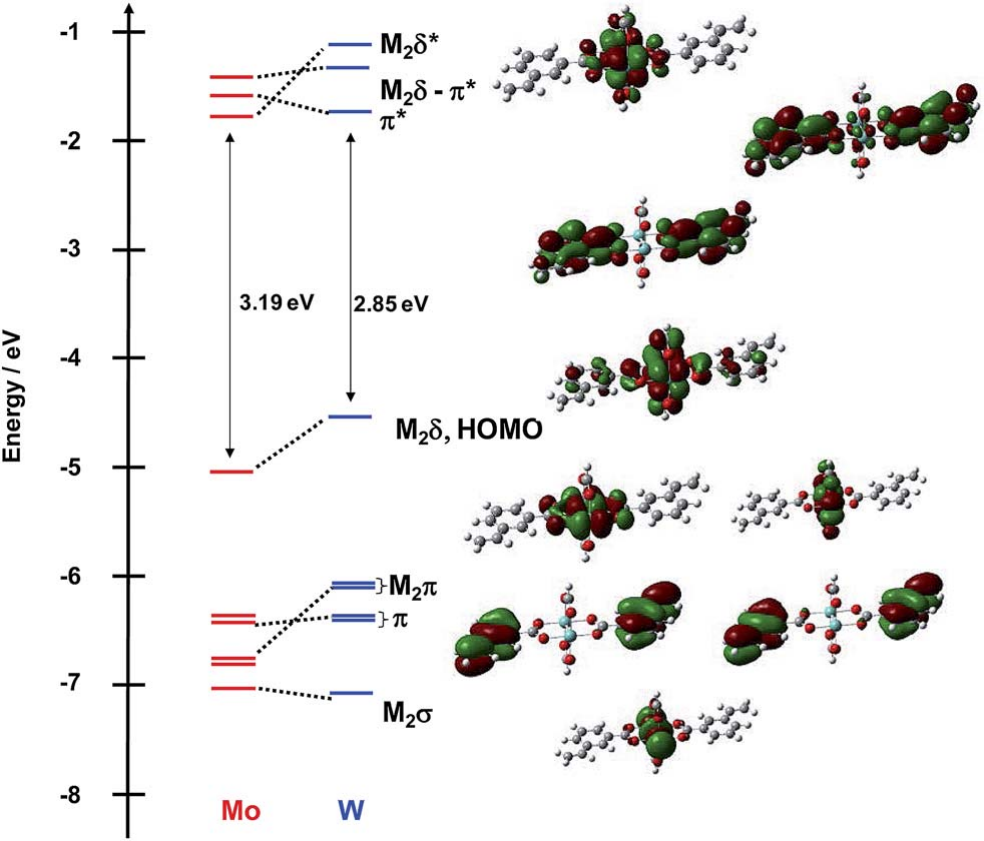
Frontier molecular orbital diagram of model compounds **1A′** and **1B′** with orbital representations of **1B′** made with GaussView 5.0 with an isovalue of 0.02.

The frontier MO energy level diagram for the *meta*-vinylbenzoate M_2_ complexes **1A′** and **1B′** is shown in Fig. 3. The HOMO for both compounds is the M_2_δ orbital with a significant degree of mixing with the ligands. The LUMO for the Mo_2_ compound is the M_2_δ*. To higher energy, the LUMO + 1 and LUMO + 2 are the symmetric and anti-symmetric combinations of the ligand π* orbitals, where the anti-symmetric combination has the correct symmetry to mix with the Mo_2_δ orbital. For the W_2_ complex, the LUMO is the symmetric π* combination and to higher energy, the anti-symmetric π* orbital is the LUMO + 1 and the M_2_δ* is the LUMO + 2.

Below the M_2_δ HOMO lie two ligand–π orbitals then two M_2_π and finally the M_2_σ for **1A′**. For the W_2_ complex **1B′**, the order of the ligand–π and M_2_π orbitals are reversed. The difference in ordering of the Lπ and M_2_π orbitals follows from the higher W_2_- *vs.* Mo_2_-based orbitals by ∼0.5 eV. The HOMO–LUMO gap for the tungsten compound tracks with the difference in M_2_δ orbital energy and is ∼0.5 eV smaller than that in the Mo_2_ analog.

**Fig. 4:**
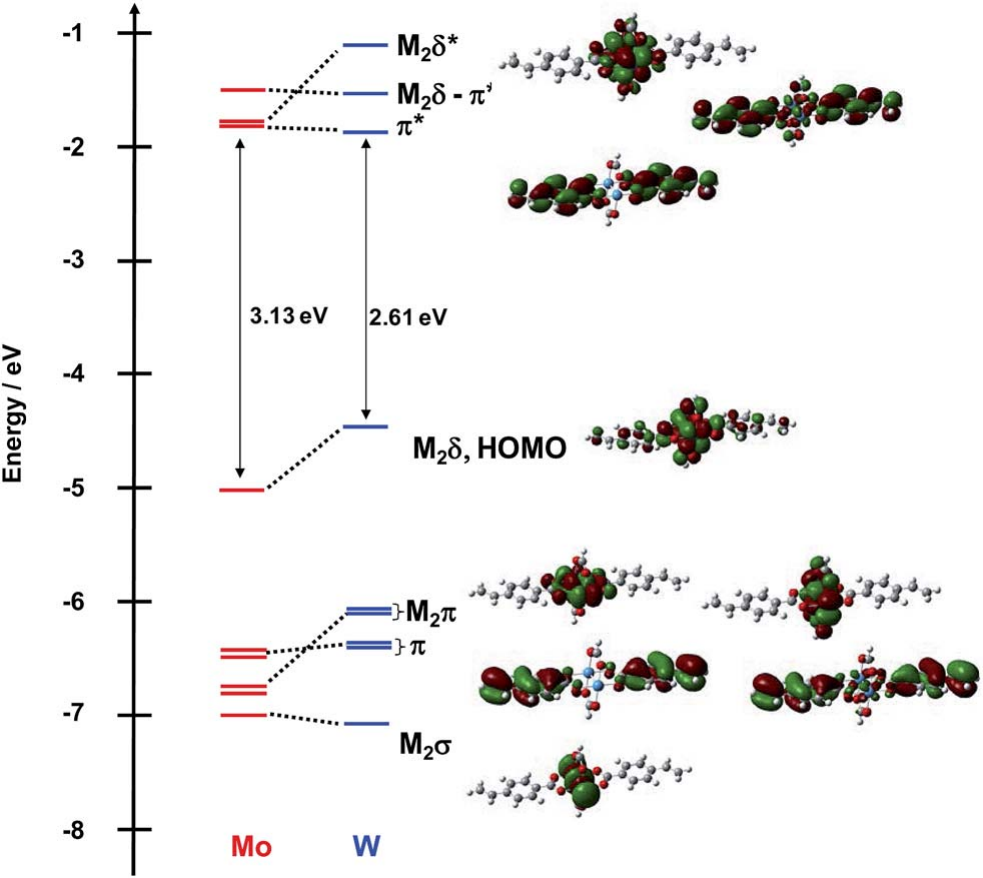
Frontier molecular orbital diagram of model compounds **2A′** and **2B′** with orbital representations of **2B′** made with GaussView 5.0 with an isovalue of 0.02.

A similar comparison of the frontier orbitals of **2A′** and **2B′** can be made and is shown in Fig. 4. Qualitatively, the orbitals are similar except for the ligand π orbitals. For this series of complexes, the vinyl substituent is in conjugation with the M_2_δ and the carboxylate core raises the energy of the occupied ligand π orbitals and lowers the energy of the ligand π* orbitals. In the Mo_2_ complex, the symmetric π* orbital is lowered below the M_2_δ* making it the LUMO. This is responsible for the reduced HOMO–LUMO gap in this series of compounds relative to **1A′** and **1B′**.

The splitting between the two combinations of π* orbitals is a representation of the electronic coupling present and the values for these compounds are listed in Table 1. Between the complexes of the same metal, the splitting does not change significantly; however, upon going from Mo_2_ to W_2_, the magnitude of the splitting increases due to the increased electronic coupling from greater mixing of the W_2_δ orbital with the ligand π*.

**Table 1:** Calculated energy splitting between the in-phase and out-of-phase combinations of π* orbitals

Compound	Δ*E*/eV
**1A′**	0.25
**1B′**	0.35
**2A′**	0.24
**2B′**	0.33
**3A′**	0.12
**4A′**	0.18

**Fig. 5:**
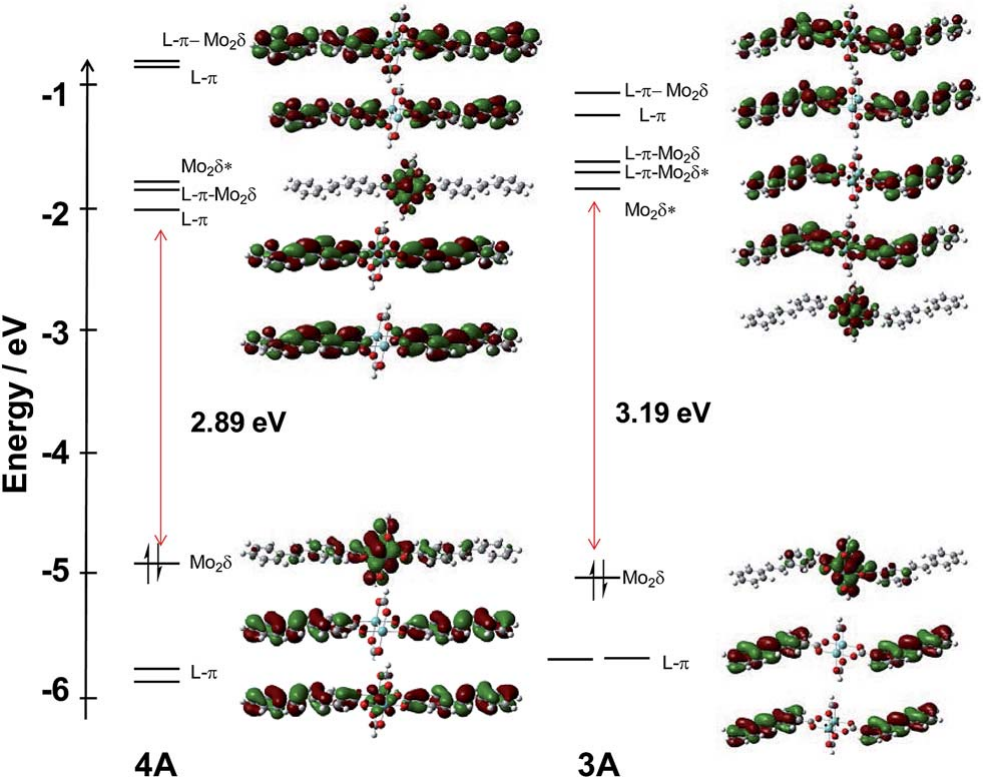
Frontier MO energy level diagram of model compounds **3A′** (right) and **4A′** (left) with orbital representations made with GaussView 5.0 with an isovalue of 0.02.

For compounds **3A′** and **4A′**, the frontier MO energy level diagrams are shown in Fig. 5. The MO diagram for compound **4A′** is very similar to its precursor **2A′**. The addition of the phenyl unit extends the π-conjugation, raises the energy of the π-orbitals, and lowers the energy of the π* orbitals. This additionally causes the secondary effect of raising the energy of the Mo_2_δ orbital. Overall, this results in a reduction of the HOMO–LUMO gap to 2.89 eV. The splitting between the two π* combinations is reduced to 0.18 eV.

For compound **3A′**, the orbital diagram is more complicated. The HOMO of **3A′** is the Mo_2_δ with some admixture from the ligands and the LUMO is the Mo_2_δ* orbital. The π system of the vinylbenzoate ligand has been perturbed relative to the parent compound **1A′**. The symmetric and anti-symmetric combinations of the Lπ* are the LUMO + 1 and LUMO + 2, respectively. Upon addition of the phenyl group, there is little energetic modification of the π* orbitals; however, there is a mixing of the Mo_2_δ* orbital with the symmetric π* combination that is not normally present in M_2_ tetracarboxylates. Further, there is character from the outer phenyl ring in this orbital, but this does not result in a change in the energy of the orbital. In addition, there are two new π* orbitals to higher energy as a result of the extended ring system in **3A′**. Below the HOMO, there is a degenerate pair of ligand π orbitals that are shifted significantly higher relative to **1A′**, –5.7 eV *vs.* –6.5 eV.

### Electronic absorption and emission spectroscopy

**Fig. 6:**
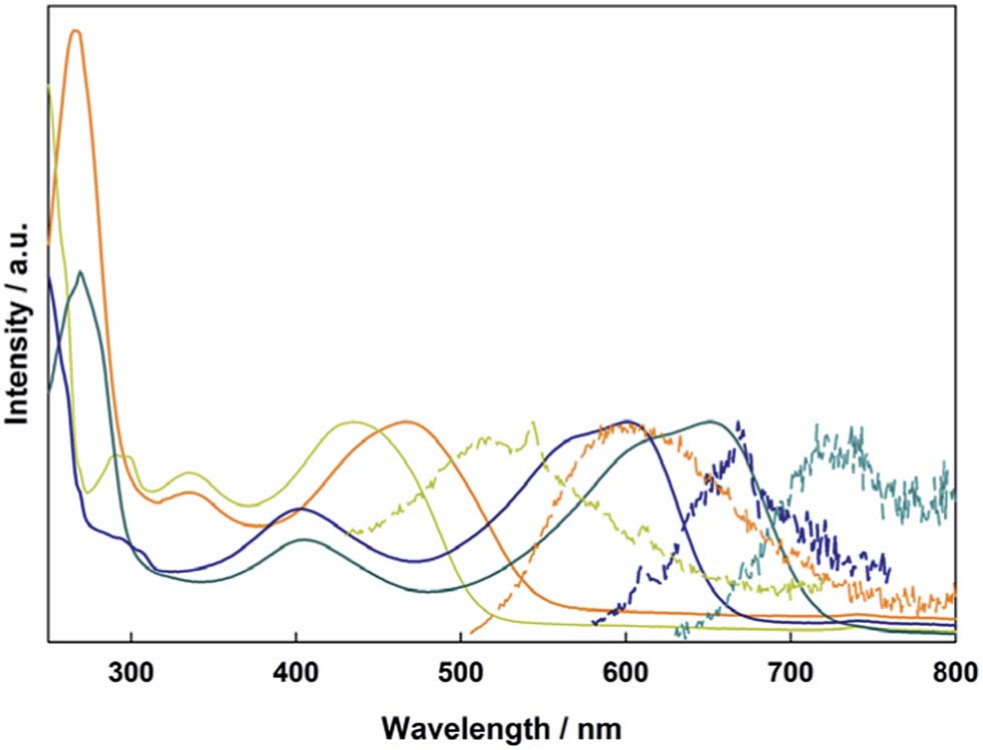
Electronic absorption (solid) and emission (dashed) of compounds **1A** (yellow), **1B** (blue), **2A** (orange), and **2B** (green) in THF.

The electronic absorption spectra of **1A**, **1B**, **2A**, and **2B** in THF are shown in Fig. 6. To higher energy, the electronic spectra of all compounds feature a ligand centered transition that is not sensitive to the metal, but is sensitive to the position of the vinyl group, with the *para*-substitution shifting the absorption to lower energy relative to the *meta*-substitution. In the Mo_2_ compounds **1A** and **2A**, there is a metal-to-ligand charge transfer from the Mo_2_δ orbital to the T^i^PB carboxylate at 330 nm. For the W_2_ compounds **1B** and **2B**, this transition occurs at 405 nm due to the raised energy of the δ orbital. Further to lower energy is the fully allowed ^1^MLCT transition from the M_2_δ orbital to the vinylbenzoate π* orbital. The observed energetic trends in the absorption maxima match those predicted by the TD-DFT calculations for the two ligand sets and two different metal centers. A table comparing the experimental and calculated absorptions can be found in the ESI.†

This MLCT transition is sensitive to both the selection of metal and the substitution pattern of the vinyl group on the benzoate ligand. For **1A**, **2A**, **1B**, and **2B** it occurs at 437 nm, 468 nm, 601 nm, and 651 nm, respectively. This trend can be easily understood by the locations of the M_2_δ and vinylbenzoate π* orbitals from the electronic structure calculations. The ^1^MLCT energy is lower for the W_2_ compounds (**1B** and **2B**) relative to Mo_2_ compounds (**1A** and **2A**) due to the raised energy of the W_2_δ orbital. The compounds with 4-vinylbenzoate (**2A** and **2B**) as the ligand occur at lower energy due to the lower π* orbital relative to 3-vinylbenzoate (**1A** and **1B**).

Also shown in Fig. 6 are the UV-visible emission spectra in THF for the four compounds described above. Upon irradiation into the MLCT absorption, these compounds show fluorescence from their ^1^MLCT states and the energy of the emission correlates with the energy of the ^1^MLCT absorption described above. The Mo_2_ compounds **1A** and **2A** also show phosphorescence from their lowest energy triplet state centered at 1100 nm, which is typical for Mo_2_ tetracarboxylates, and corresponds to emission from the ^3^MoMoδδ* state. Upon cooling to 77 K, the emission notably increases in intensity and a vibronic progression corresponding to the Mo–Mo stretch (∼350 cm^–1^) becomes visible. Compounds **1B** and **2B** show broad ^1^MLCT emissions in the visible and NIR extending to 1200 nm. There is no evidence of any phosphorescence from either W_2_ complex. The NIR emission data can be seen in the ESI Fig. S1.†

**Fig. 7:**
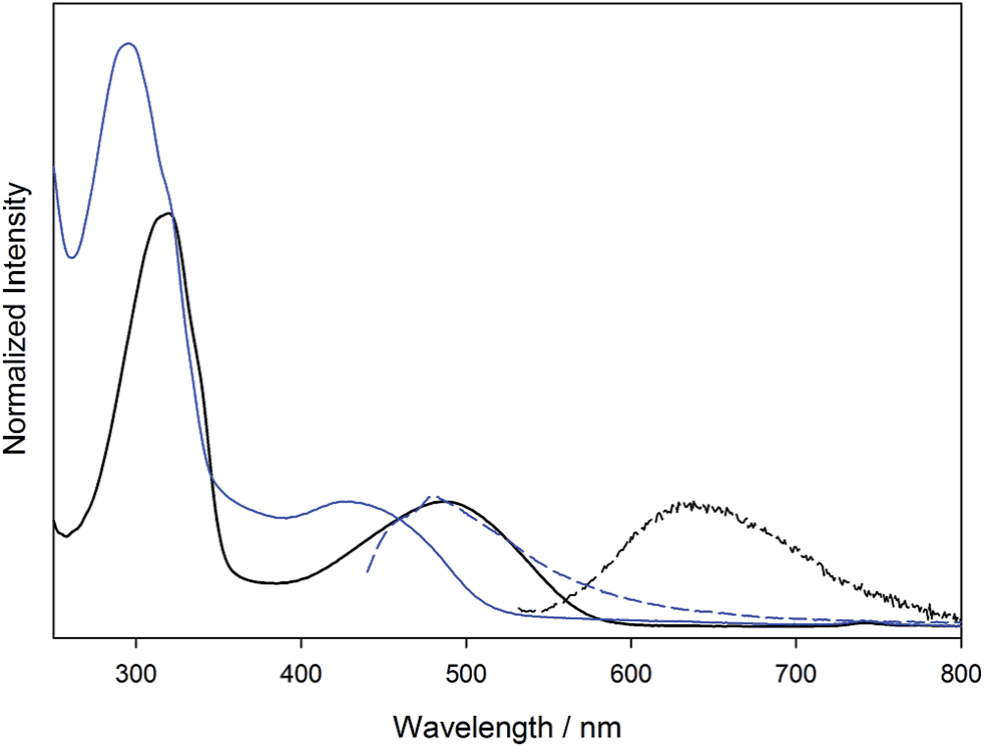
Electronic absorption (solid) and emission (dashed) for **3A** (blue) and **4A** (black) in THF.

The electronic absorption and emission of **3A** and **4A** were taken in THF and can be seen in Fig. 7. These compounds show the same gross features as their parent Mo_2_ compounds **1A** and **2A**. For **3A**, the addition of the phenyl substituent shifts the π–π* absorption to lower energy (295 nm) compared to the parent complex **1A**. However, the energy of the ^1^MLCT absorption remains at almost the same wavelength (430 nm). For **4A**, the π–π* absorption (319 nm) is shifted to lower energy than that in **2A** (267 nm) and in **3A** (295 nm). The ^1^MLCT energy shifts to lower energy (487 nm) relative to the parent **2A** (467 nm) and to the *meta*-substituted **3A** (430 nm).

**Fig. 8:**
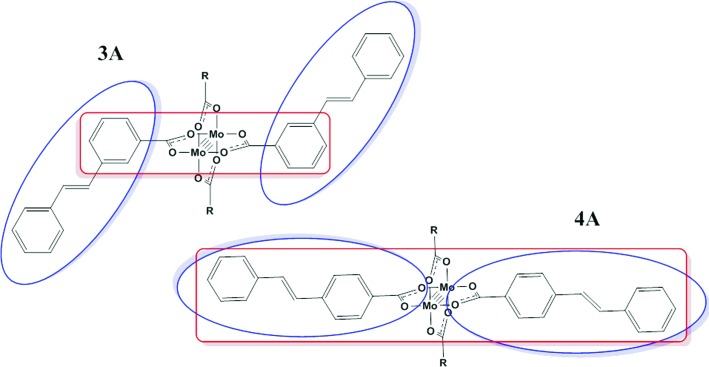
Location of the representative absorption bands π–π* (blue) and MLCT (red) present in **3A** and **4A**, where R = 2,4,6-triisopropylphenyl.

The change in the energy of the π–π* and MLCT absorptions in **3A** and **4A** can be understood in terms of their substitution pattern. For **3A**, the *meta* substitution pattern determines the outer phenyl-vinyl moiety is not in conjugation with the carboxylate or the Mo_2_ center. The π–π* is then located principally on the phenyl-vinyl-phenyl subunit and has no contribution from the carboxylate. The *meta*-substitution has no effect on the MLCT and therefore it behaves as if the ligand is simply a benzoate. For **4A**, the entire ligand phenyl-vinyl-phenyl-CO_2_ is involved in the π–π* transition making it occur at slightly lower energy than in **3A**. Since the substitution pattern is *para* in **4A**, the MLCT spans the entire ligand and not just the simple benzoate as in **3A**. This is shown pictorially in Fig. 8 with colored boxes denoting the subunits of the molecule involved in each transition for **3A** and **4A**.

Both **3A** and **4A** show emission from their ^1^MLCT states upon irradiation in THF that correlates energetically with the MLCT absorption (Fig. 7), similar to the emission of the parent compounds **1A** and **2A**. Both compounds also show emission in the NIR centered around 1100 nm. Upon cooling, vibronic features (∼350 cm^–1^) become visible and are associated with the Mo–Mo stretch. Combined, this leads to the assignment of ^3^MoMoδδ* emission as seen in the parent compounds **1A** and **2A**. The NIR emission spectra can be seen in the ESI Fig. S2.†

### Transient absorption spectroscopy

**Fig. 9:**
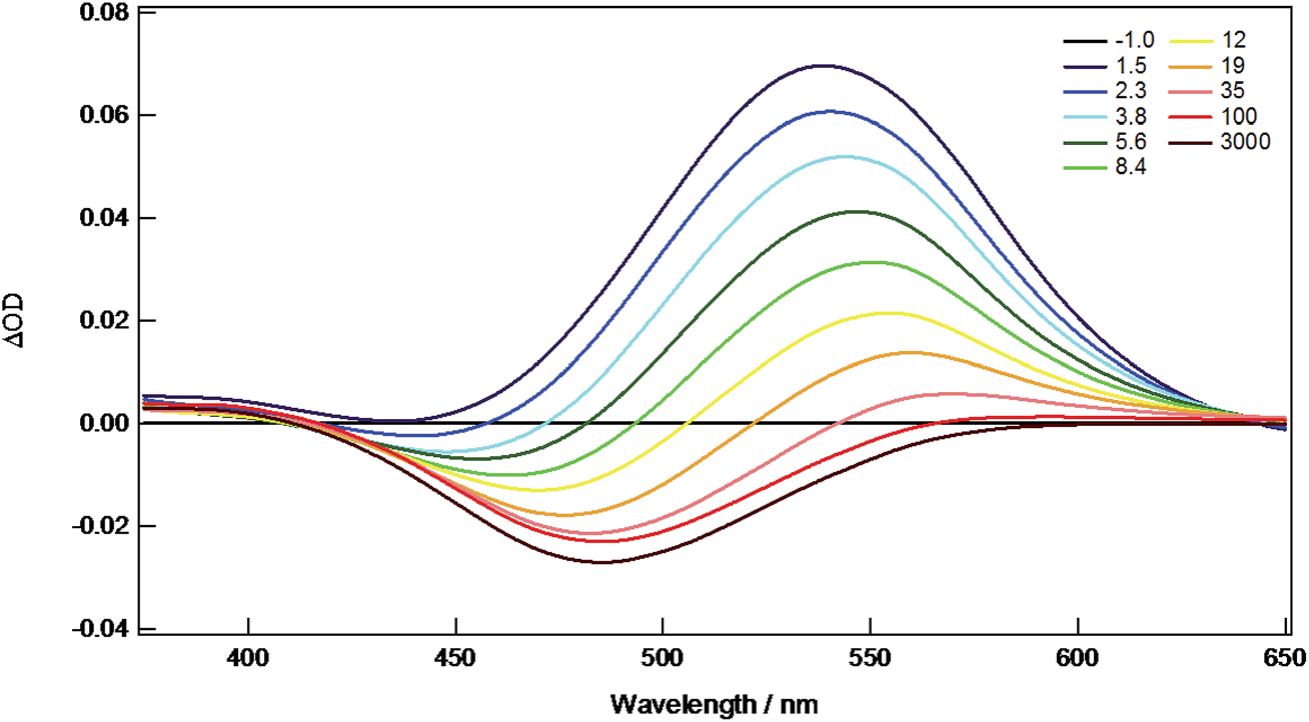
fsTA spectrum of **4A** in THF, *λ*_ex_ = 335 nm.

The new compounds **1A**, **1B**, **2A**, **2B**, **3A**, and **4A** were all investigated by nanosecond (ns) and femtosecond (fs) transient absorption (TA) spectroscopy in THF solution to monitor the singlet and triplet states. The fsTA spectra of **4A** are shown as representative in Fig. 9. Here, there is a strong absorption centered on 540 nm present from the singlet state that decays within ∼9 ps to the triplet state represented by a persistent bleach at 480 nm that lasts beyond 3 ns. The kinetics of the singlet states were measured for all compounds and the lifetimes fall within 0.5–10 ps, typical for ^1^MLCT S_1_ states, and are listed in Table 2. The other TA spectra and kinetics are shown in ESI Fig. S3–S13.†

**Table 2:** Lifetimes and assignments of S_1_ and T_1_ excited states determined from fs and ns transient absorption measurements

	S_1_ lifetime	S_1_ assignment	T_1_ lifetime	T_1_ assignment
**1A**	3.0 ps	^1^MLCT	89.5 μs	^3^δδ*
**1B**	0.5 ps	^1^MLCT	3–10 ns	^3^MLCT
**2A**	2.4 ps	^1^MLCT	64.3 μs	^3^δδ*
**2B**	1.7 ps	^1^MLCT	∼30 ns	^3^MLCT
**3A**	5.7 ps	^1^MLCT	58.1 μs	^3^δδ*
**4A**	9.2 ps	^1^MLCT	63 μs	^3^δδ*

Estimation of the lifetimes of the triplet states for all compounds were made from the nsTA spectra primarily from the decay of the ground state bleaches. For the molybdenum compounds, the lifetimes are 50–90 μs. Spectra and kinetics are shown in ESI Fig. S14–S21.† This long lifetime coupled with the NIR emission is indicative of a ^3^MoMoδδ* T_1_ state. The W_2_ complexes **1B** and **2B** show much faster dynamics. For **1B**, there is evidence of a long-lived component on the fsTA experiment but no features on the nsTA experiment. This indicates a triplet state with a lifetime of 3–10 ns which is between the resolutions of the two instruments. Compound **2B** shows weak features on the nsTA experiment that decay in ∼30 ns. Spectra and kinetics are shown in ESI Fig. S22 and S23.† Previously, the T_1_ state of W_2_T^i^PB_4_ was determined to be ^3^WWδδ* with a lifetime of ∼1 μs and phosphorescence from this state was observed around 860 nm.^16^ The much faster triplet lifetimes of **1B** and **2B** coupled with the lack of phosphorescence lead to the assignment of a ^3^MLCT T_1_ state in the W_2_ complexes.

Upon examination of the S_1_ excited state lifetimes, some trends are revealed. The Mo_2_ complexes, **1A** and **2A**, show longer-lived S_1_ states when compared with their W_2_ counterparts, **1B** and **2B**. This may be attributed to the heavy atom effect and the larger spin orbit coupling of tungsten as compared to molybdenum. The shorter S_1_ lifetime of **3A** compared with **4A** may be a result of the mixing of the Mo_2_δ* as evidenced by the electronic structure calculations, *vida supra*, suggesting a more efficient pathway for conversion to the δδ* state.

### Time-resolved infrared spectroscopy

In order to examine charge distribution in the excited states of these molecules, compounds **2A**, **2B**, and **4A** have been studied by fs time resolved infrared (TRIR) spectroscopy. In principle, the vibrational frequency of the vinyl group should be sensitive to the excited state electron distribution and therefore be able to provide insight into charge localization; however, this has not been shown to be the case. With the assistance of the electronic structure calculations employing time-dependent DFT, the dominant features are attributable to the phenyl ring stretches and the carboxylate stretching modes.

**Fig. 10:**
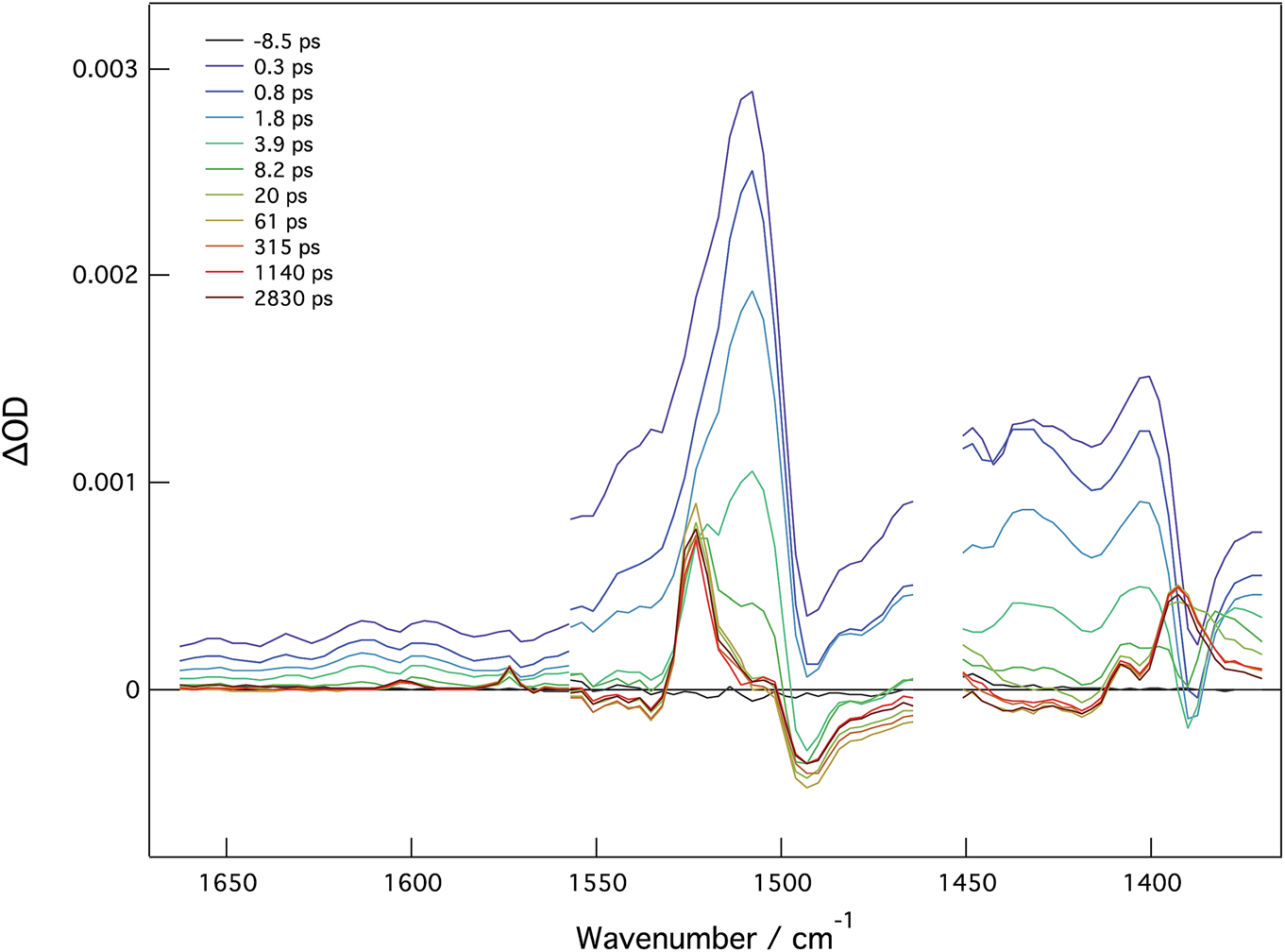
fsTRIR spectra of **2A** in THF, *λ*_ex_ = 515 nm.

For **2A**, the TRIR spectra are shown in Fig. 10 and the kinetics can be seen in Fig. S24.† Present is a large transient at 1525 cm^–1^ that has been assigned to phenyl ring stretching mode. This feature decays with a lifetime of 3.5 ps, which is similar to the singlet lifetime found in the fsTA experiment for this compound. At long times, there are features that are associated with the carboxylate stretches and have previously been assigned as characteristic of the ^3^MoMoδδ* state. Specifically, the band at 1525 cm^–1^ is assignable to *ν*_as_(CO_2_) of the T_1_ state that, relative to the ground state, has one less electron in the δ orbital which is involved in back-bonding to the CO_2_π*, thus causing the vibration to increase in energy.^17^

For **2B**, the phenyl stretching mode is present as a transient in the singlet state at 1546 cm^–1^ and carboxylate stretches appear at 1481 cm^–1^ and 1405 cm^–1^ (ESI Fig. S25†). From the decay of these features, the singlet lifetime is estimated to be 2 ps, which is effectively the same lifetime that was determined by fsTA spectroscopy. There are several ligand vibrations at 1532 cm^–1^, 1503 cm^–1^, and 1405 cm^–1^ indicative of a long-lived triplet state as seen in the fsTA. Apparent is the lack of features associated with the δδ* state. This, coupled with the lack of phosphorescence is consistent with a ^3^MLCT T_1_ state.

**Fig. 11:**
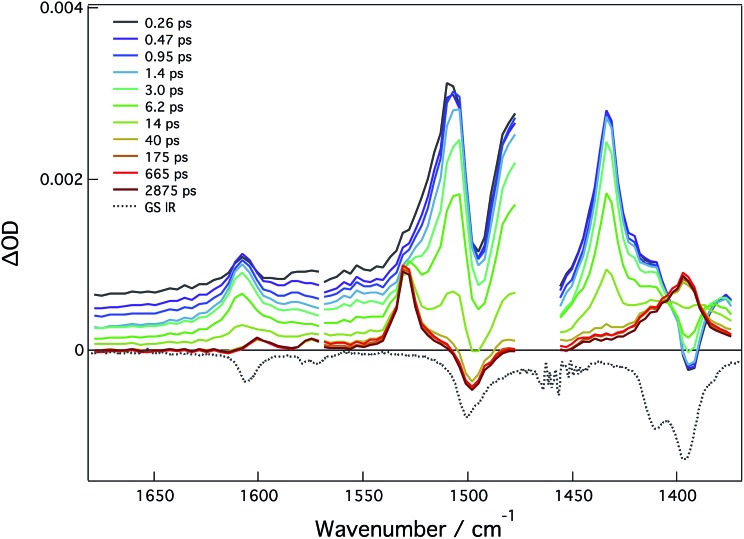
Ground state IR (black dashed) and fsTRIR of **4A** taken in THF, *λ*_ex_ = 515 nm.

The ground state IR and fsTRIR of compound **4A** were taken in THF and are largely dominated by –CO_2_ and phenyl ring stretching modes, see Fig. 11. In the ground state, the bands at 1607 cm^–1^ and 1570 cm^–1^ are assigned to phenyl ring stretching modes. The band at 1495 cm^–1^ is assigned to the *ν*_as_(CO_2_) and the band at 1380 cm^–1^ to the *ν*_s_(CO_2_). Upon excitation, there are four transients, which correspond to the two *ν*(CO_2_) stretching modes and the two phenyl ring stretching modes. From these features, the lifetime of the ^1^MLCT is estimated to be 10 ps, which is in good agreement with the fsTA data (ESI Fig. S26†). At longer times, the characteristic modes of the ^3^MoMoδδ* state are present indicating this as the T_1_ state.

**Fig. 12:**
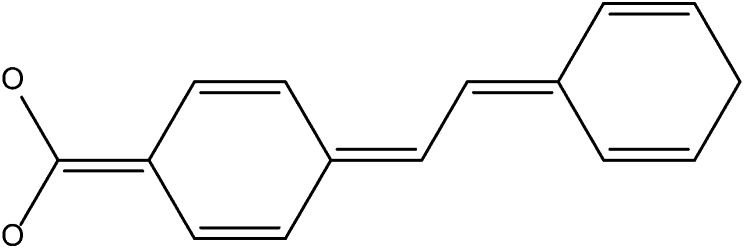
Quinoidal structure of the supporting ligand of **4A** in the ^1^MLCT state.

The lack of clear vinyl features in the TRIR spectra preclude the assignment of excited state distribution. Instead of a discrete vinyl mode, the C=C stretch couples to the breathing modes of the phenyl rings as would be expected in the quinoidal form of the ligand in **4A**, Fig. 12.

Previously, metal complexes bearing *trans*-stilbene or derivatives of *trans*-stilbene as ligands have been shown to undergo *cis*/*trans* isomerization upon photoexcitation. The complexes, fac-Re(CO)_3_(bpy)(X), where X = 1,2-bis(4-pyridyl)ethylene [bpe] or 4-styrylpyridine [stpy], were shown to undergo photoisomerization.^18^ These complexes were excited at 313 nm into a transition with IL and MLCT character, where photoisomerization then took place with quantum yields in the range of 0.003–0.46 depending upon the solvent. It was proposed that this isomerization occurs through a ^3^p* intermediate, where the olefin is twisted.^19^ In a related case, the bpe ligand was used to bridge two Re centers or Re and Os together in complexes of the form, [(CO)_3_(phen)Re(*trans*-bpe)Re(phen)(CO)_3_]^2+^ and [(phen)Re(CO)_3_(*trans*-bpe)Os(trpy)(bpy)]^3+^ respectively.^20^ In the bis Re case, photoisomerization occurred despite the bulky groups on either end of the bpe ligand. Conversely, no photoisomerization was observed for the mixed metal Re/Os which was attributed to a low lying MLCT state of the osmium unit that quenches the chemical reaction.

In this regard, it is worth exploring the potential for isomerization in **4A**. After photoexcitation, we observe rapid internal conversion (<1 ps) to the ^1^MLCT S_1_ state, where the excited electron resides in a π* orbital and the hole remains on the Mo_2_δ orbital. This MLCT state lies lower in energy than the π–π* states responsible for isomerization thereby preventing photoisomerization from occurring similar to that seen in the mixed Re/Os case discussed above.

## Conclusions

Several new vinylbenzoate supported M_2_ bis–bis complexes were prepared including two compounds, **3A** and **4A**, by the Heck cross coupling reactions involving **1A** and **2A**, respectively. Based on this work the prospect of performing carbon–carbon cross coupling reactions on the periphery of M_2_ complexes in the preparation of extended structures containing M_2_ quadruply bonded units looks promising. The photophysical properties of the new compounds have been explored, but the vinyl groups did not prove to be viable IR reporters of excited localization or delocalization across the M_2_ center. Alternatively, the use of C≡X functionalities, where X = N, O, or CR, has proved largely successful in the determination of charge localization or delocalization in M_2_ complexes.^13,21,22^

## Experimental section

### General methods

All reactions were performed under 1 atm of UHP argon using standard Schlenk or glovebox techniques. Solvents were dried and distilled from the appropriate drying agents and then degassed prior to use. Solvents were stored over 4 Å molecular sieves in flasks with Kontes tops. Manipulations of the studied compounds were performed under nitrogen inside a glovebox or under argon on a Schlenk line.

NMR spectra were recorded on a 400 MHz Bruker DPX Advance 400 spectrometer or a 500 MHz DPX Advance 500 spectrometer. All ^1^H NMR chemical shifts are in parts per million (ppm) relative to the protio impurity in THF-d_8_ at 1.73 ppm.

High resolution matrix-assisted laser desorption ionization time-of-flight (MALDI-TOF) mass spectra were obtained on a Bruker Microflex mass spectrometer for **1A** and **2A** and calibrated using an internal standard. All other samples were analyzed on a Bruker ultrafleXtreme mass spectrometer run in positive mode. The measurements for **1B**, **2B**, **3A**, and **4A** were standardized using 9 peptides with molecular weights ranging from 750 g mol^–1^ to 3100 g mol^–1^. These peptides were supported in a matrix of HCCA. Dithranol was used as the matrix. Samples were prepared by adding a solution of the matrix to the solid sample. This was then spotted on the plate for analysis. Also, the observed isotope pattern for each sample has been correlated to the predicted pattern based on the given formulation.

3-Vinylbenzoic acid and 4-vinylbenzoic acid were purchased from Alfa Aesar and used as received. Mo_2_T^i^PB_4_ and W_2_T^i^PB_4_ were synthesized from known procedures.^16,23^ Iodobenzene and palladium(ii) acetate were purchased from Sigma Aldrich. Iodobenzene was degassed by freeze–pump–thaw and stored over 4 Å molecular sieves in a Kontes top flask under argon.

### Electronic structure calculations

Density functional theory was used to perform electronic structure calculations on model complexes utilizing the Gaussian 09 suite of programs.^24^ The Becke three parameter correlation functional combined with the Lee, Yang, and Parr exchange functional was employed (B3LYP).^25,26^ Model complexes were optimized in the gas phase to a minimum as confirmed by the lack of imaginary frequencies in a vibrational analysis. For C, H, and O the 6-31g* basis set was used. For Mo and W, the Stuttgart/Dresden energy-consistent pseudopotential (SDD) was used to represent the core orbitals and the SDD basis set.^27^

Formate was used instead of T^i^PB in order to simplify the calculations and save on computational resources. This substitution allows the complexes to obtain an idealized symmetry higher than that with T^i^PB. The complexes were idealized to the highest symmetry possible while still containing an inversion center. A correction factor of 0.96 was applied to the calculated vibrational frequencies to correlate them with experimentally observed frequencies.

Visualizations of the molecular orbitals was performed with GaussView 5.0 with an isovalue of 0.02.

### Electronic absorption and emission

Steady-state electronic absorption and UV-visible emission measurements were carried out with 1.0 × 1.0 cm quartz cuvettes equipped with Kontes stopcocks. Electronic absorption spectra at room temperature were recorded using a Perkin-Elmer Lambda 900 spectrometer in THF solution.

Fluorescence measurements were made on a SPEX Fluoromax-2 spectrofluorometer in the UV-visible region in THF solution. The compounds were irradiated into their MLCT absorption bands. Emission measurements in the near-infrared region were performed in J. Young NMR tubes at room temperature and 77 K in 2-methyl THF. Spectra were recorded on a home-built instrument equipped with a germanium detector. Samples were irradiated into their MLCT bands. On the NIR setup, this was done with either 405 nm (**1A–4A**) or 658 nm (**1B**, **2B**). Samples were prepared with an absorbance of <0.3.

### Crystallographic information

Single crystals of **1A** and **2A** were isolated as yellow and orange blocks, respectively, and handled under a pool of fluorinated oil. Examination of the diffraction pattern was done on a Nonius Kappa CCD diffractometer with Mo Kα radiation. All work was done at 150 K using an Oxford Cryosystems Cryostream Cooler. Data integration was done with Denzo, and scaling and merging of the data was done with Scalepack.^28^

The structures were solved by the direct methods program in SHELXS-13.^29^ Full-matrix least-squares refinements based on F^2^ were performed in SHELXL-13,^29^ as incorporated in the WinGX package.^30^ For each methyl group, the hydrogen atoms were added at calculated positions using a riding model with *U*(H) = 1.5*U*_eq_ (bonded carbon atom). The rest of the hydrogen atoms were included in the model at calculated positions using a riding model with *U*(H) = 1.2*U*_eq_ (bonded atom). Neutral atom scattering factors were used and include terms for anomalous dispersion.^31^ One isopropyl group in the T^i^PB moiety of **1A** was disordered over two locations. This disorder was modeled using similarity restraints as well as rigid bond restraints to ensure reasonable bond lengths and anisotropic displacement parameters. The occupancy of these two parts was allowed to refine on a free variable leading to an occupancy of ∼54% in the major component.

Compound **2A** had disorder in the *p*-vinyl moiety. This disorder was handled similarly to that in **1A** and the occupancy of the major component refined to be ∼50%. After final refinement of **2A** several residual ‘Q’ peaks remained in the difference map and were unable to be adequately modeled as solvent. To remove this electron density the SQUEEZE^32^ protocol of the PLATON^33^ suite of programs was used to exclude 45 electrons from a void of 250 Å^3^. This electron density likely corresponds to disordered hexanes used in crystallization of **2A**.†

### Time-resolved spectroscopy

Nanosecond TA was performed on samples in 1 × 1 cm square quartz cuvettes with Kontes stopcocks.^34^ Measurements were made on a home-built instrument pumped by a frequency-doubled (532 nm) or frequency tripled (355 nm) Spectra-Physics GCR-150 Nd:YAG laser (fwhm ≈ 8 ns, ∼5 mJ per pulse). The power at the sample was set to 100 mW Signal from a Hamamatsu R928 photomultiplier tube was processed with a Tektronics 400 MHz oscilloscope (TDS 380). For **1A**, **1B**, **2B**, and **3A** the samples were excited with 355 nm and for **2A** and **4A** the samples were excited at 532 nm with laser power at the sample of 100 mW.

Femtosecond transient absorption experiments were performed with a Ti:sapphire and regenerative amplifier combination (1 kHz, 50 fs full width at half-maximum) that has been previously described.^35^ Samples were prepared with absorbances of ∼0.3–0.8 in a 1.0 mm quartz cuvette with Kontes top. Excitation power at the sample was 1–2 μJ. Spectra collected underwent wavelength calibration and group velocity dispersion corrections.

Time-resolved infrared^36^ spectroscopy experiments, previously described, were performed with a Ti:sapphire and regenerative amplifier combination (1 kHz, 50 fs fwhm). Samples of **2A**, **2B** and **4A** were prepared with an absorbance of ∼1 at *λ*_max_ in THF solution. A PerkinElmer semi-demountable cell with a 0.1 mm Teflon spacer between 4 mm CaF_2_ windows was used to provide an airtight sample chamber. The compounds **2A**, **2B**, and **4A** were excited with a laser power of ∼1 μJ at 515 nm, 515 nm and 675 nm, respectively.

In general, kinetics were fit to a sum of exponential decay terms, *S*(*t*) = ∑_*i*_ *A*_*i*_ exp(–1/*τ*_*i*_) + *C*, using Igor Pro 6.0 or SigmaPlot 12.0, where *A*_*i*_ is the amplitude, *τ* is the lifetime, and *C* is an offset. Error bars for the lifetimes are reported as standard errors of the exponential fits.

### Synthesis

Mo_2_(T^i^PB)_2_(3VB)_2_ [**1A**]. Mo_2_T^i^PB_4_ [520 mg, 0.440 mmol] and 3-vinylbenzoic acid [130 mg, 0.878 mmol] were added to a Schlenk flask and toluene [40 mL] was added. The flask stirred for 3 days after which a yellow precipitate had formed. The solvent was reduced *in vacuo* to 5 mL and hexanes [30 mL] added to fully precipitate the product. The yellow solid was then filtered and washed with further hexanes [2 × 30 mL] and dried under vacuum [325 mg, 75%]. ^1^H NMR (THF-d_8_): *δ*_H_ (400 MHz) 8.43 (s, 2H), 8.26 (d, 2H, *J*_HH_ = 8 Hz), 7.67 (d, 2H, *J*_HH_ = 8 Hz), 7.54 (t, 2H, *J*_HH_ = 8 Hz), 6.99 (d, 2H, *J*_HH_ = 8 Hz), 6.88 (dd, 2H, *J*_HH_ = 11 Hz, 17 Hz), 5.89 (d, 2H, *J*_HH_ = 11 Hz), 5.31 (d, 2H, *J*_HH_ = 11 Hz), 3.04 (sep, 4H, *J*_HH_ = 6.9 Hz), 2.87 (sep, 2H, *J*_HH_ = 7.7 Hz), 1.23 (d, 12H, *J*_HH_ = 7.7 Hz), 1.02 (d, 24H, *J*_HH_ = 6.9 Hz). MALDI-TOF calculated monoisotopic MW for C_50_H_60_Mo_2_O_8_: 981. Found: 981.6 (M^+^). UV-vis (THF): 437 nm, 337 nm, 296 nm.

W_2_(T^i^PB)_2_(3VB)_2_ [**1B**]. W_2_T^i^PB_4_ [245 mg, 0.184 mmol] and 3-vinylbenzoic acid [51 mg, 0.331 mmol] were weighed out and added to a Schlenk flask. Toluene [15 mL] was added and the reaction stirred for 5 days. The solvent was then removed *in vacuo* and the residue suspended in hexanes [15 mL]. This was then filtered and washed further with hexanes [2 × 30 mL]. The desired product was then isolated as a maroon powder after drying under vacuum [148 mg, 70%]. ^1^H NMR (THF-d_8_): *δ*_H_ (400 MHz) 8.22 (s, 2H), 8.05 (d, 2H, *J*_HH_ = 8 Hz), 7.52 (t, 2H, *J*_HH_ = 8 Hz), 7.44 (d, 2H, *J*_HH_ = 8 Hz), 6.98 (s, 4H, *J*_HH_ = 8 Hz), 6.84 (dd, 2H, *J*_HH_ = 11 Hz, 17 Hz), 5.89 (d, 2H, *J*_HH_ = 11 Hz), 5.30 (d, 2H, *J*_HH_ = 17 Hz), 2.84 (m, 6H), 1.21 (d, 12H, *J*_HH_ = 7 Hz), 0.97 (d, 24H, *J*_HH_ = 7.5 Hz). MALDI-TOF calculated monoisotopic MW for C_50_H_60_W_2_O_8_: 1156.33. Found: 1156.55 (M^+^). UV-vis (THF): 601 nm, 563 nm, 401 nm.

Mo_2_(T^i^PB)_2_(4VB)_2_ [**2A**]. Mo_2_T^i^PB_4_ [405 mg, 0.343 mmol] and 4-vinylbenzoic acid [102 mg, 0.689 mmol] were added to a Schlenk flask. Toluene [30 mL] was added and the flask stirred for 3 days after which, an orange precipitate had formed. The solvent was reduced *in vacuo* to 5 mL toluene and hexanes [30 mL] was added to fully precipitate the product. The product was then filtered and washed with hexanes [2 × 30 mL] then dried under vacuum to leave an orange powder [270 mg, 80%]. ^1^H NMR (THF-d_8_): *δ*_H_ (400 MHz) 8.33 (d, 4H, 8.8 Hz), 7.65 (d, 4H, *J*_HH_ = 8.8 Hz), 6.99 (s, 4H), 6.88 (dd, 2H, *J*_HH_ = 11.5 Hz, 17.2 Hz), 5.89 (d, 2H, *J*_HH_ = 11.2 Hz), 5.36 (d, 2H, *J*_HH_ = 17.5 Hz), 3.04 (sep, 4H, *J*_HH_ = 7.0 Hz), 2.87 (sep, 2H, *J*_HH_ = 7.2 Hz), 1.23 (d, 12H, *J*_HH_ = 6.8 Hz), 1.03 (d, 24H, *J*_HH_ = 6.8 Hz). MALDI-TOF calculated monoisotopic MW for C_50_H_60_Mo_2_O_8_: 981. Found: 981.6 (M^+^). UV-vis (THF): 467 nm, 335 nm, 267 nm.

W_2_(T^i^PB)_2_(4VB)_2_ [**2B**]. W_2_T^i^PB_4_ [255 mg, 0.180 mmol] and 4-vinylbenzoic acid [49 mg, 0.368 mmol] were weighed out and added to a Schlenk flask. To this was added 15 mL of toluene and the flask stirred for 5 days. Upon mixing, a blue solution and blue precipitate formed. The solvent was removed *in vacuo* and the residue stirred in hexanes, filtered and washed with further hexanes [2 × 30 mL]. The blue solid was then dried under vacuum [169 mg, 69%]. ^1^H NMR (THF-d_8_): *δ*_H_ (400 MHz) 8.10 (d, 4H, 7 Hz), 7.65 (d, 4H, *J*_HH_ = 7 Hz), 6.98 (s, 4H), 6.89 (dd, 2H, *J*_HH_ = 11.1 Hz, 17.7 Hz), 5.82 (d, 2H, *J*_HH_ = 17.2 Hz), 5.36 (d, 2H, *J*_HH_ = 11.2 Hz), 2.84 (m, 6H), 0.97 (d, 24H, *J*_HH_ = 7 Hz). MALDI-TOF calculated monoisotopic MW for C_50_H_60_W_2_O_8_: 1156.33. Found: 1156.57 (M^+^). UV-vis (THF): 652 nm, 605 nm, 405 nm, 269 nm.

Mo_2_(T^i^PB)_2_(3VB-Ph)_2_ [**3A**]. Mo_2_(T^i^PB)_2_(3VB)_2_ [ 100 mg, 0.101 mmol], and palladium(ii) acetate [2 mg, 0.009 mmol] were added to a Kontes top flask and dissolved in THF [5 mL]. To this was added iodobenzene [312 mg, 1.53 mmol] in THF [3 mL] and Et_3_N [3 mL]. The flask was sealed and then heated to 75 °C for 72 hours after which the yellow color became more intense. The solution was filtered through a bed of celite and the solvent removed *in vacuo*. The residue was then washed with hexanes [2 × 10 mL], dissolved in a minimum amount of THF [2 mL] and the product precipitated as a yellow powder with hexanes [10 mL]. The yellow powder was then filtered and dried under vacuum, [80 mg, 70%]. ^1^H NMR (THF-d_8_): *δ*_H_ (500 MHz) 8.560 (s, 2H), 8.27 (d, 2H, *J*_HH_ = 7.6 Hz), 7.82 (d, 2H, *J*_HH_ = 7.7 Hz), 7.58 (m, 6H), 7.29–7.36 (m, 8H), 7.23 (t, 2H, *J*_HH_ = 7.3 Hz), 6.99 (s, 4H), 3.06 (sep, 4H, *J*_HH_ = 6.9 Hz), 2.86 (sep, 2H, *J*_HH_ = 6.9 Hz), 1.20 (d, 12H, *J*_HH_ = 6.9 Hz), 1.05 (d, 24H, *J*_HH_ = 6.9 Hz). MALDI-TOF calculated monoisotopic MW for C_62_H_68_Mo_2_O_8_: 1133.30. Found: 1133.53 (M^+^). UV-vis (THF): 427 nm, 318 nm (sh), 295 nm.

Mo_2_(T^i^PB)_2_(4VB-Ph)_2_ [**4A**]. Mo_2_(T^i^PB)_2_(4VB)_2_ [200 mg, 0.202 mmol], and palladium(ii) acetate [10 mg, 0.045 mmol] were added to a Kontes top flask and dissolved in THF [5 mL]. To this was added iodobenzene [680 mg, 3.33 mmol] in THF [5 mL] and Et_3_N [6 mL]. The flask was sealed and then heated to 65 °C for 4 days after which the orange color became more intense. The solution was evaporated to dryness *in vacuo* and the residue dissolved in THF [10 mL]. This was filtered through a bed of celite and the solvent removed *in vacuo*. The residue then stirred in hexanes [10 mL] for 24 hours, filtered, and washed further with hexanes [3 × 20 mL]. Further purification involved recrystallization by vapor diffusion of hexanes into a THF solution. The orange powder was then dried under vacuum [175 mg, 75%]. ^1^H NMR (THF-d_8_): *δ*_H_ (500 MHz) 8.36 (d, 4H, *J*_HH_ = 8.1 Hz), 7.77 (d, 4H, *J*_HH_ = 8.2 Hz), 7.62 (d, 4H, *J*_HH_ = 8.0 Hz), 7.36 (m, 8H), 7.25 (t, 2H, *J*_HH_ = 6.7 Hz), 6.99 (s, 4H), 3.05 (sep, 4H, *J*_HH_ = 6.7 Hz), 2.86 (sep, 4H, *J*_HH_ = 6.9 Hz), 1.22 (d, 12H, *J*_HH_ = 6.8 Hz), 1.04 (d, 24H, *J*_HH_ = 6.9 Hz) MALDI-TOF calculated monoisotopic MW for C_62_H_68_Mo_2_O_8_: 1133.30. Found: 1133.55 (M^+^). UV-vis (THF): 486 nm, 318 nm.

## References

[cit1] Burroughes J. H., Bradley D. D. C., Brown A. R., Marks R. N., Mackay K., Friend R. H., Burns P. L., Holmes A. B. (1990). Nature.

[cit2] Braun D., Heeger A. J. (1992). Thin Solid Films.

[cit3] Yu G., Gao J., Hummelen J. C., Wudl F., Heeger A. J. (1995). Science.

[cit4] Hide F., DÍaz-GarcÍa M. A., Schwartz B. J., Heeger A. J. (1997). Acc. Chem. Res..

[cit5] Barbara P. F., Gesquiere A. J., Park S.-J., Lee Y. J. (2005). Acc. Chem. Res..

[cit6] Sheats J. R., Chang Y.-L., Roitman D. B., Stocking A. (1999). Acc. Chem. Res..

[cit7] Bässler H., Schweitzer B. (1999). Acc. Chem. Res..

[cit8] Halls J. J. M., Walsh C. A., Greenham N. C., Marseglia E. A., Friend R. H., Moratti S. C., Holmes A. B. (1995). Nature.

[cit9] Peng Z., Yu L. (1996). J. Am. Chem. Soc..

[cit10] Wong C. T., Chan W. K. (1999). Adv. Mater..

[cit11] Liu Y., Li Y., Schanze K. S. (2002). J. Photochem. Photobiol., C.

[cit12] Ren T. (2008). Chem. Rev..

[cit13] Brown-Xu S. E., Chisholm M. H., Durr C. B., Spilker T. F. (2013). J. Am. Chem. Soc..

[cit14] Burdzinski G. T., Chisholm M. H., Chou P.-T., Chou Y.-H., Feil F., Gallucci J. C., Ghosh Y., Gustafson T. L., Ho M.-L., Liu Y., Ramnauth R., Turro C. (2008). Proc. Natl. Acad. Sci. U. S. A..

[cit15] Albert Cotton F., Walton R. A., and Murillo C. A., Multiple bonds between metal atoms, Oxford, Oxford, 3rd edn, 2005.

[cit16] Alberding B. G., Chisholm M. H., Chou Y.-H., Gallucci J. C., Ghosh Y., Gustafson T. L., Patmore N. J., Reed C. R., Turro C. (2009). Inorg. Chem..

[cit17] Alberding B. G., Chisholm M. H., Gustafson T. L. (2011). Inorg. Chem..

[cit18] Polo A. S., Itokazu M. K., Frin K. M., de Toledo Patrocínio A. O., Murakami Iha N. Y. (2006). Coord. Chem. Rev..

[cit19] Dattelbaum D. M., Itokazu M. K., Murakami Iha N. Y., Meyer T. J. (2003). J. Phys. Chem. A.

[cit20] Itokazu M. K., Polo A. S., Iha N. Y. M. (2003). J. Photochem. Photobiol., A.

[cit21] Alberding B. G., Chisholm M. H., Gallucci J. C., Ghosh Y., Gustafson T. L. (2011). Proc. Natl. Acad. Sci. U. S. A..

[cit22] Brown-Xu S. E., Chisholm M. H., Durr C. B., Lewis S. A., Spilker T. F., Young P. J. (2014). Inorg. Chem..

[cit23] Cotton F. A., Daniels L. M., Hillard E. A., Murillo C. A. (2002). Inorg. Chem..

[cit24] Frisch M. J., Trucks G. W., Schlegel H. B., Scuseria G. E., Robb M. A., Cheeseman J. R., Scalmani G., Barone V., Mennucci B., Petersson G. A., Nakatsuji H., Caricato M., Li X., Hratchian H. P., Izmaylov A. F., Bloino J., Zheng G., Sonnenberg J. L., Hada M., Ehara M., Toyota K., Fukuda R., Hasegawa J., Ishida M., Nakajima T., Honda Y., Kitao O., Nakai H., Vreven T., Montgomery Jr J. A., Peralta J. E., Ogliaro F., Bearpark M., Heyd J. J., Brothers E., Kudin K. N., Staroverov V. N., Kobayashi R., Normand J., Raghavachari K., Rendell A., Burant J. C., Iyengar S. S., Tomasi J., Cossi M., Rega N., Millam J. M., Klene M., Knox J. E., Cross J. B., Bakken V., Adamo C., Jaramillo J., Gomperts R., Stratmann R. E., Yazyev O., Austin A. J., Cammi R., Pomelli C., Ochterski J. W., Martin R. L., Morokuma K., Zakrzewski V. G., Voth G. A., Salvador P., Dannenberg J. J., Dapprich S., Daniels A. D., Farkas Ö., Foresman J. B., Ortiz J. V., Cioslowski J. and Fox D. J., Gaussian 09, Revision A.1, Gaussian, Inc., Wallingford CT, 2009.

[cit25] Miehlich B., Savin A., Stoll H., Preuss H. (1989). Chem. Phys. Lett..

[cit26] Lee C., Yang W., Parr R. G. (1988). Phys. Rev. B: Condens. Matter Mater. Phys..

[cit27] Chisholm M. H., D'Acchioli J. S., Hadad C. M. (2006). J. Cluster Sci..

[cit28] Otwinowski Z. and Minor W., Methods in Enzymology, Vol. 276: Macromolecular Crystallography, Part A, Academic Press, 1997.

[cit29] Sheldrick G. (2008). Acta Crystallogr., Sect. A: Found. Crystallogr..

[cit30] Farrugia L. (1999). J. Appl. Crystallogr..

[cit31] Wilson A. J. C. and Geist V., International Tables for Crystallography, Volume C, Kluwer Academic Publishers, Dordrecht, 1992.

[cit32] Van Der Sluis P., Spek A. L. (1990). Acta Crystallogr., Sect. A: Found. Crystallogr..

[cit33] Spek A. L. (1990). Acta Crystallogr., Sect. A: Found. Crystallogr..

[cit34] Byrnes M. J., Chisholm M. H., Gallucci J. A., Liu Y., Ramnauth R., Turro C. (2005). J. Am. Chem. Soc..

[cit35] Burdzinski G., Hackett J. C., Wang J., Gustafson T. L., Hadad C. M., Platz M. S. (2006). J. Am. Chem. Soc..

[cit36] Wang J., Burdzinski G., Kubicki J., Platz M. S. (2008). J. Am. Chem. Soc..

