# Supplementary material for: MM quadruply bonded complexes supported by vinylbenzoate ligands: synthesis, characterization, photophysical properties and application as synthons
Source: Chem Sci. 2015 Jan 13;6(3):1780–91. doi: 10.1039/c4sc02542c (PMC4583209; doi:10.1039/c4sc02542c)
Supplement: Supplementary file 1 [file SC-006-C4SC02542C-s001.pdf]

Electronic Supplementary Material (ESI) for Chemical Science.
This journal is © The Royal Society of Chemistry 2014

SUPPORTING INFORMATION

MM Quadruply Bonded Complexes Supported by Vinylbenzoate Ligands: Synthesis, Characterization, Photophysical Properties and Application as Synthons

Samantha E. Brown-Xu, Malcolm H. Chisholm,* Christopher B. Durr, Thomas F. Spilker and Philip J. Young

Table of Contents

S1 – S2…………………………………………………………………………………...NIR Emission

S3 – S13……………………………………………………………fs-Transient Absorption and kinetics

S14 – S24…………………………………………………………..ns-Transient Absorption and kinetics

S25………………………………………………………………………fs-Time Resolved Infrared of **2B**

S26……………………………………………………………………………………Kinetic trace of **4A**

S27 – S32………………………………………………………^{1}H NMR in d$_8$-THF at room temperature

S33 – S38…………………………………………………………High resolution MALDI-TOF Mass Spec

S39……………………………………...Comparison of experimental and theoretical electronic transitions

S40……………………………………………Crystallographic Data Collection Parameters for **1A** and **1B**

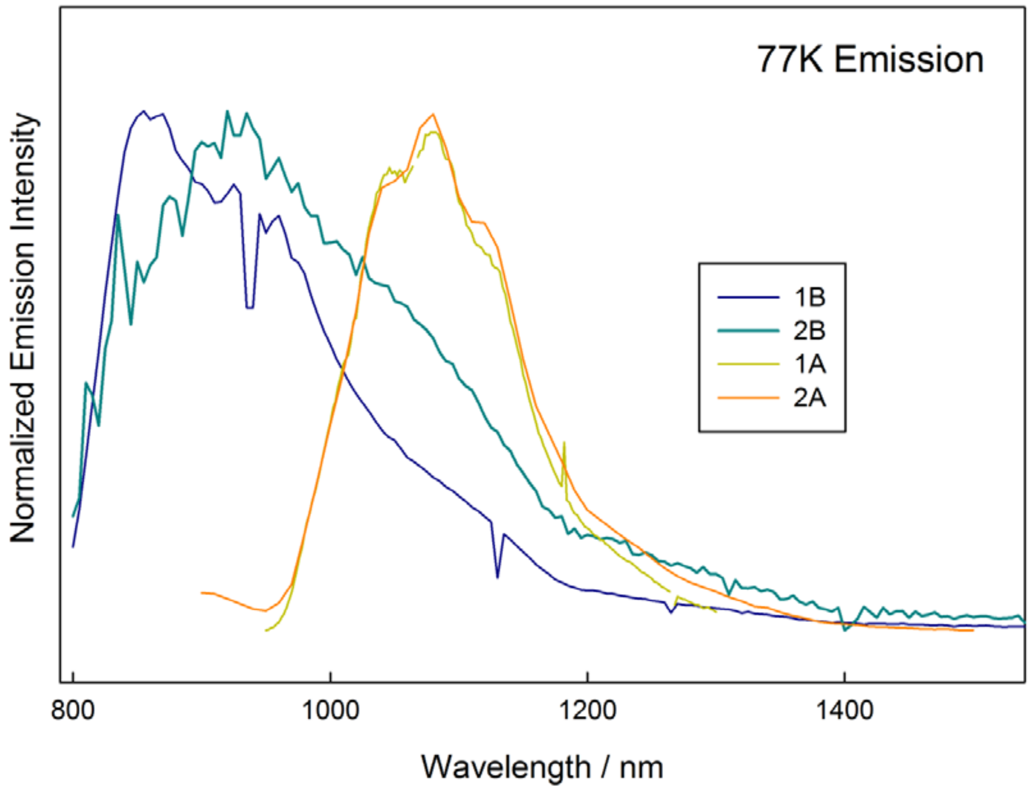

Figure S1. NIR emission of **1A**, **1B**, **2A**, and **2B** in 2-MeTHF at 77K.

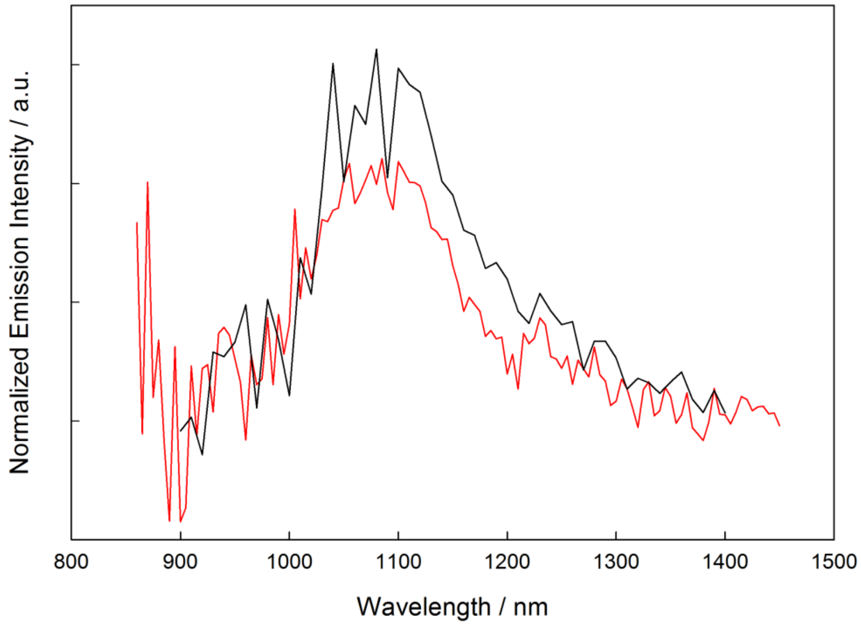

Figure S2. NIR emission of **3A** (black) and **4A** (red) in 2Me-THF at room temperature.

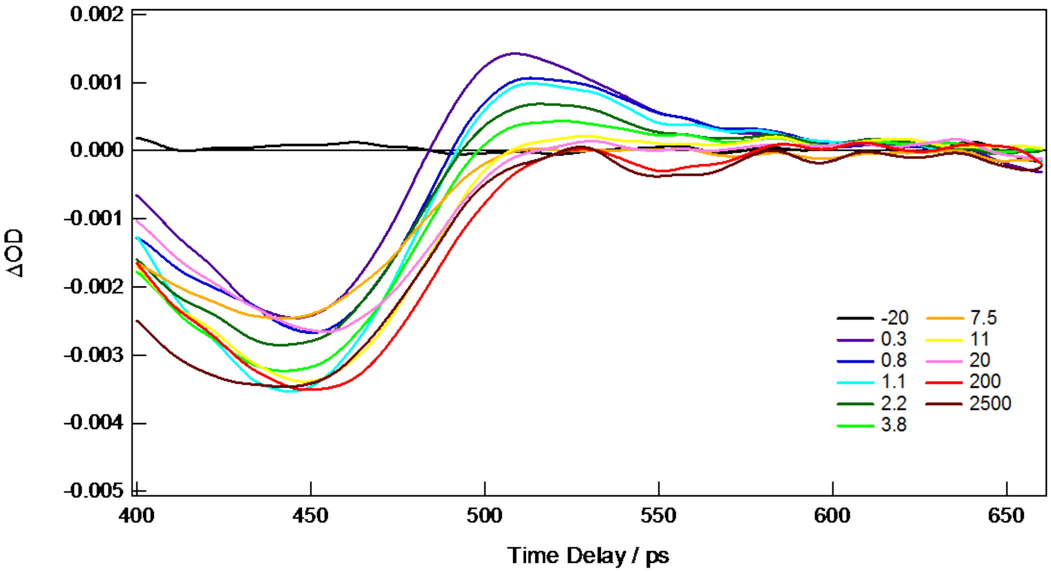

5 Figure S3. fsTA spectra of **1A** in THF with excitation at 350 nm.

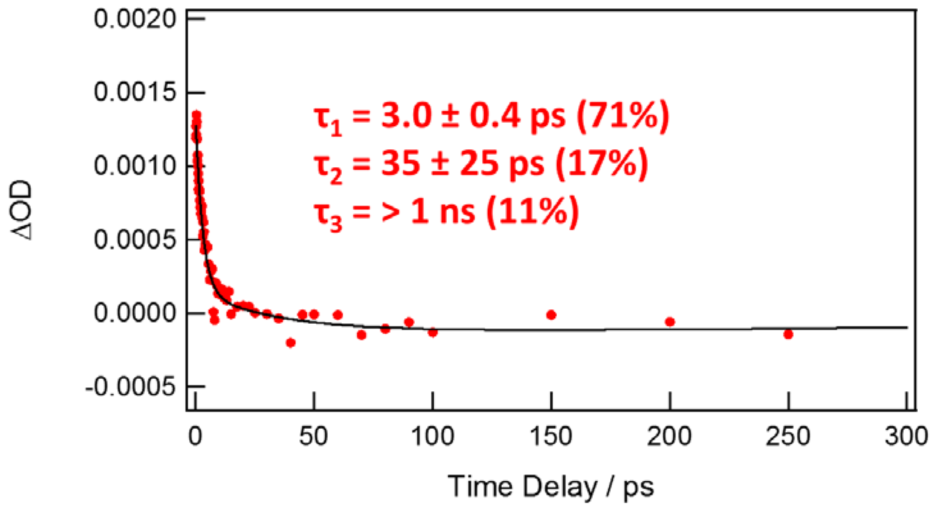

Figure S4. Kinetic trace from fsTA spectra of **1A** taken at 520 nm.

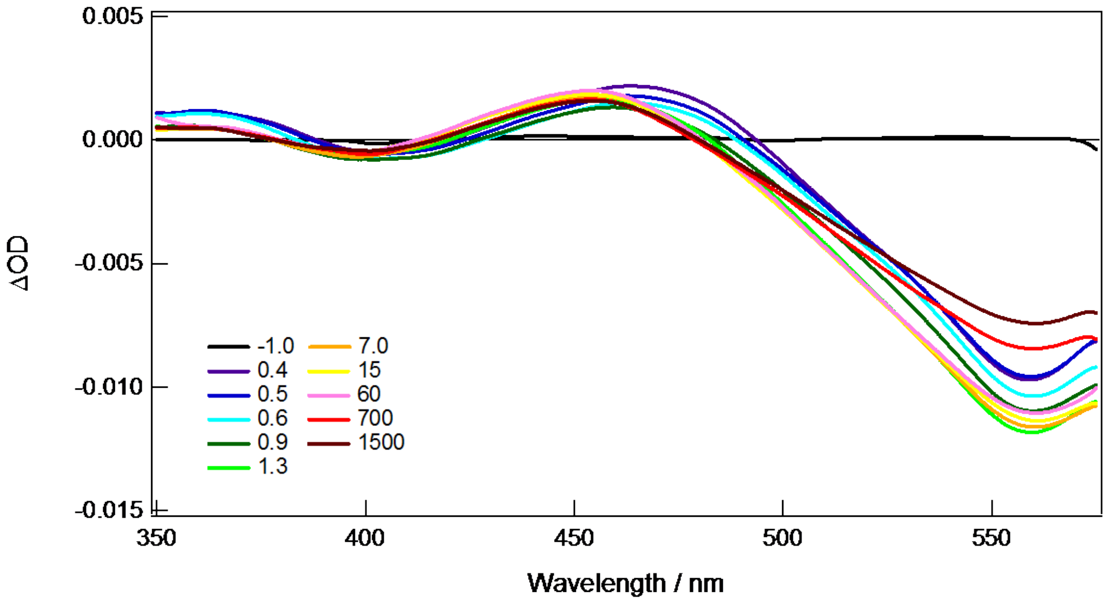

Figure S5. fsTA spectra of **1B** in THF with excitation at 600 nm.

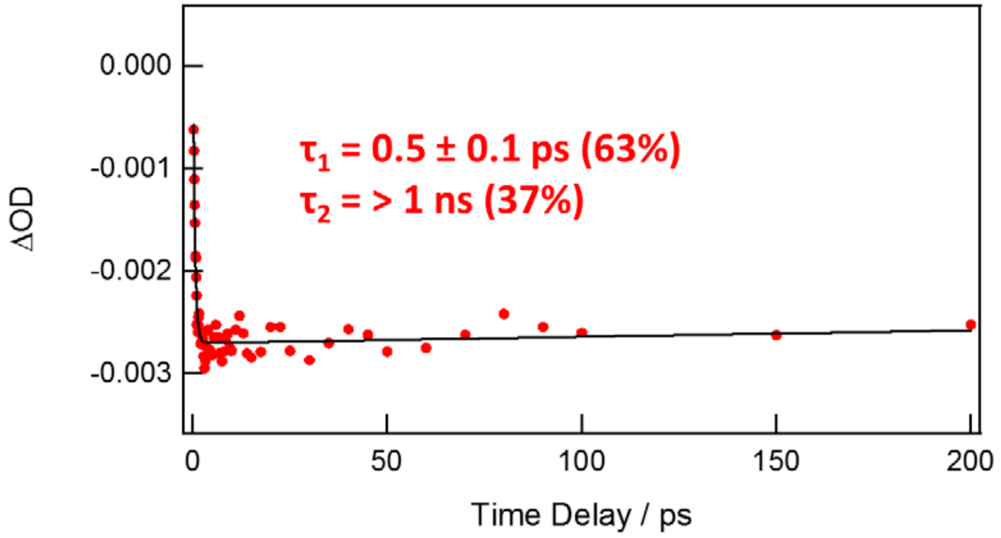

Figure S6. Kinetic trace from fsTA spectra of **1B** taken at 500 nm.

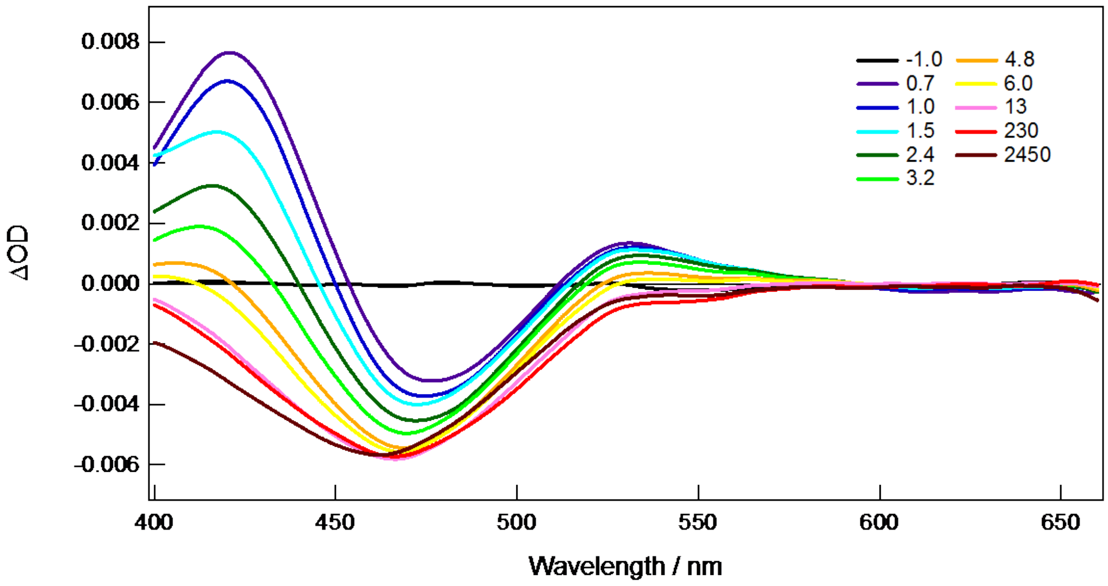

Figure S7. fsTA spectra of **2A** in THF with excitation at 350 nm.

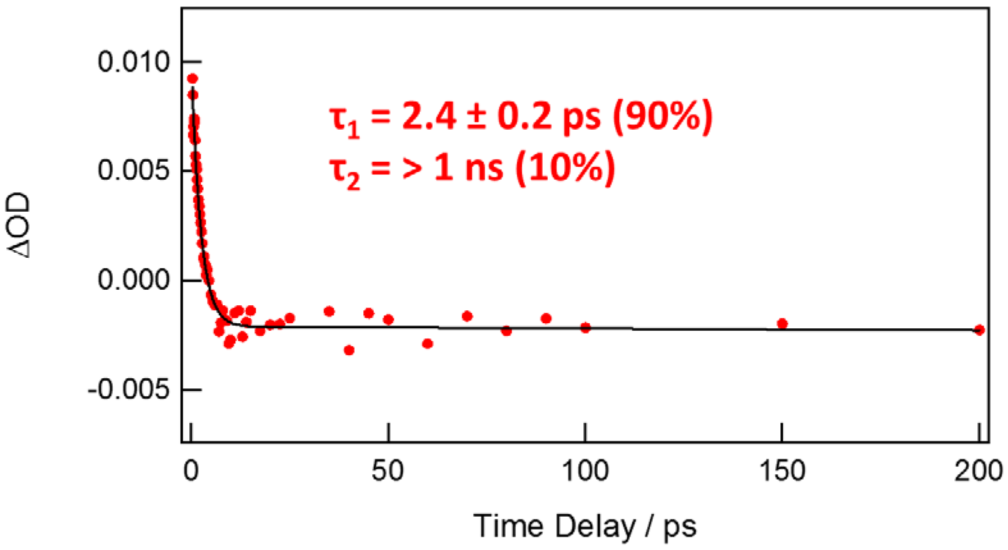

Figure S8. Kinetic trace from fsTA spectra of **2A** taken at 425 nm.

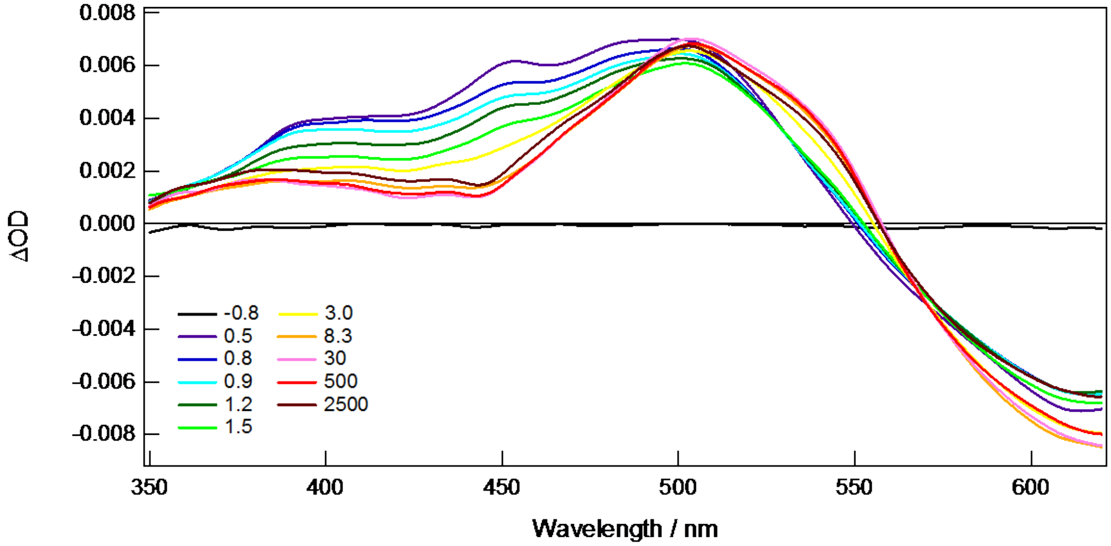

Figure S9. fsTA spectra of **2B** in THF with excitation at 675 nm.

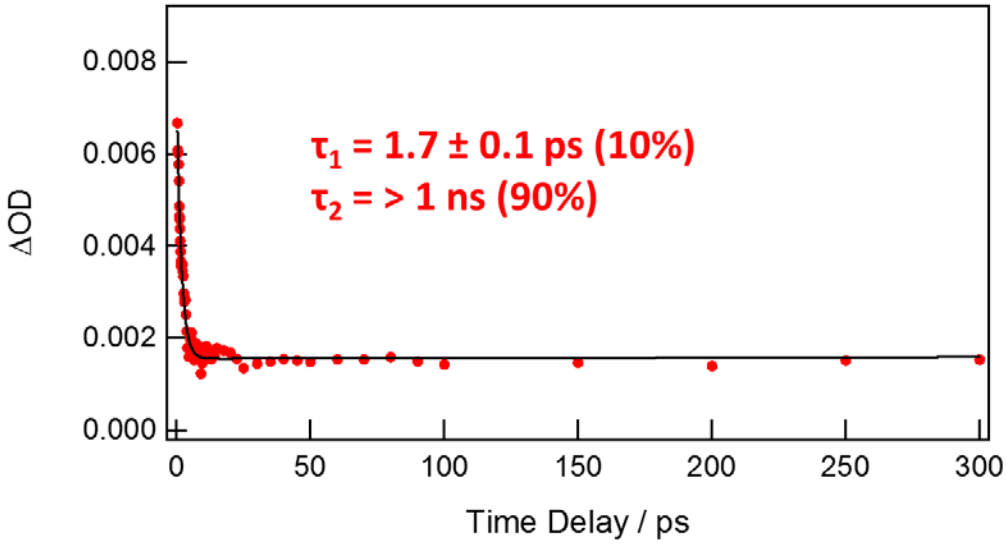

Figure S10. Kinetic trace from fsTA spectra of **2B** taken at 450 nm.

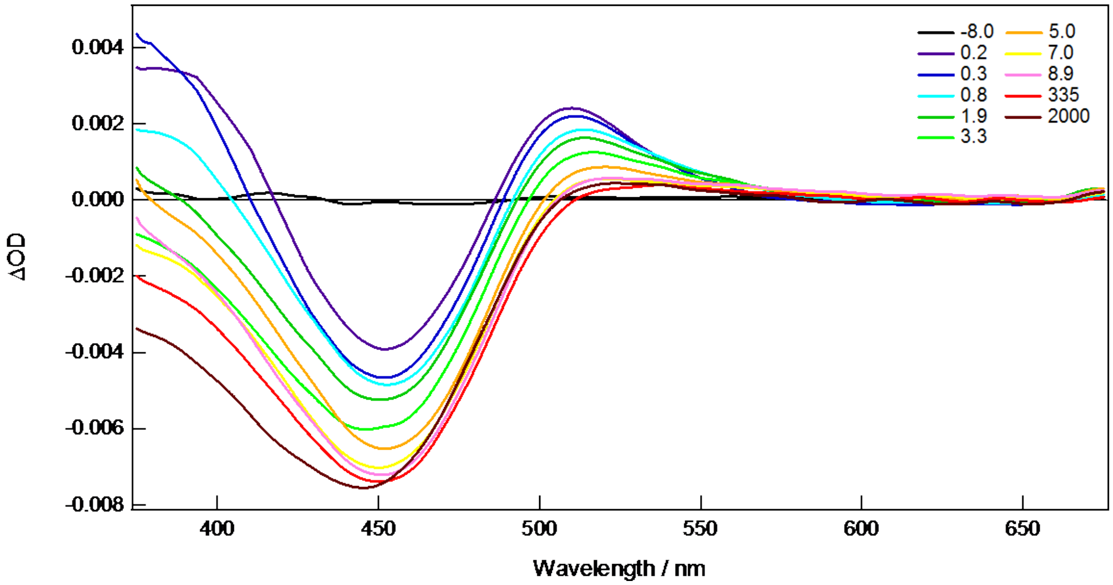

Figure S11. fsTA spectra of **3A** in THF with excitation at 350nm.

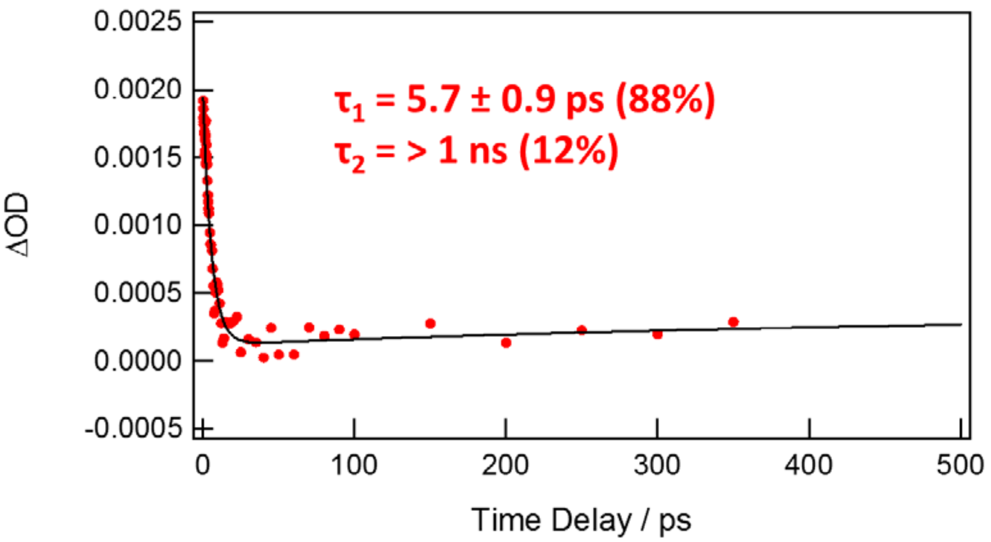

Figure S12. Kinetic trace from fsTA spectra of **3A** taken at 520 nm.

5

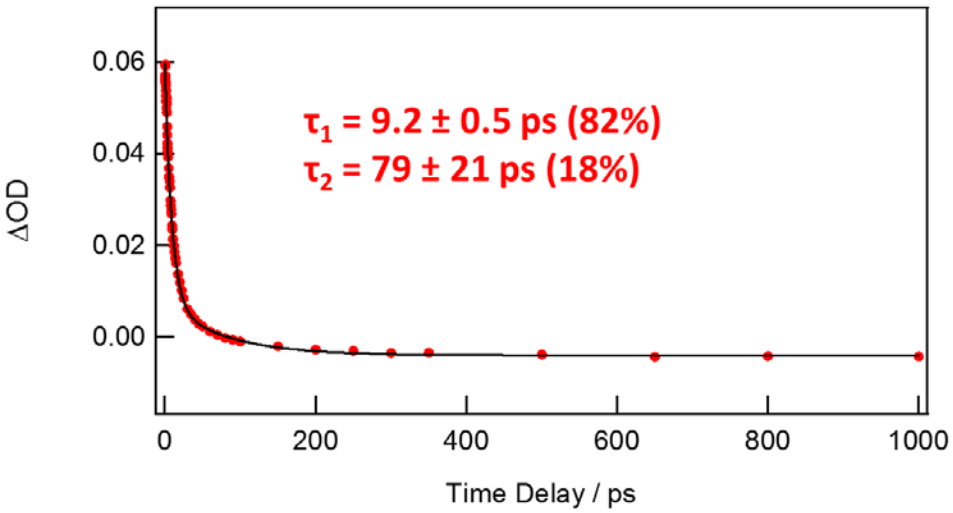

Figure S13. Kinetic trace from fsTA spectra of **4A** taken at 560 nm.

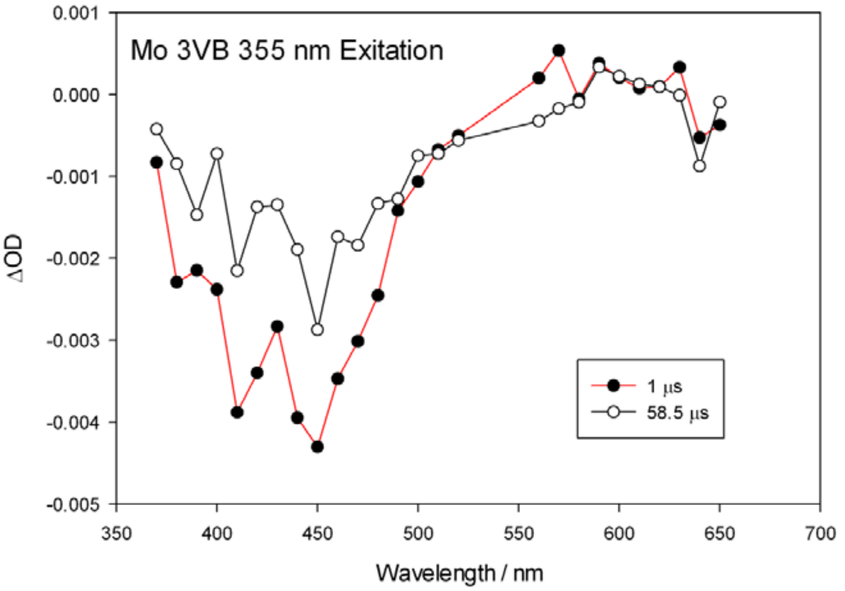

Figure S14. nsTA spectra of **1A** in THF with excitation at 355 nm.

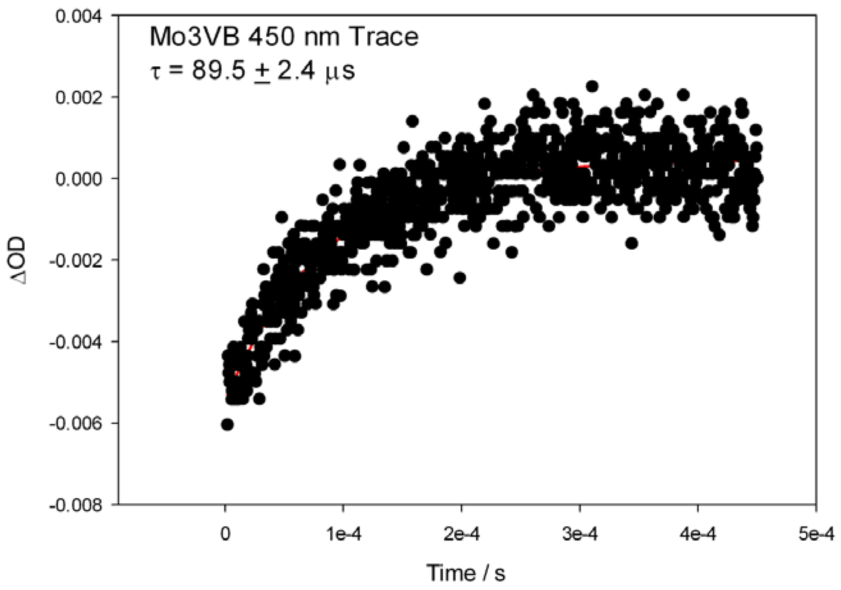

Figure S15. Kinetic trace from nsTA spectra of **1A** taken at 540 nm.

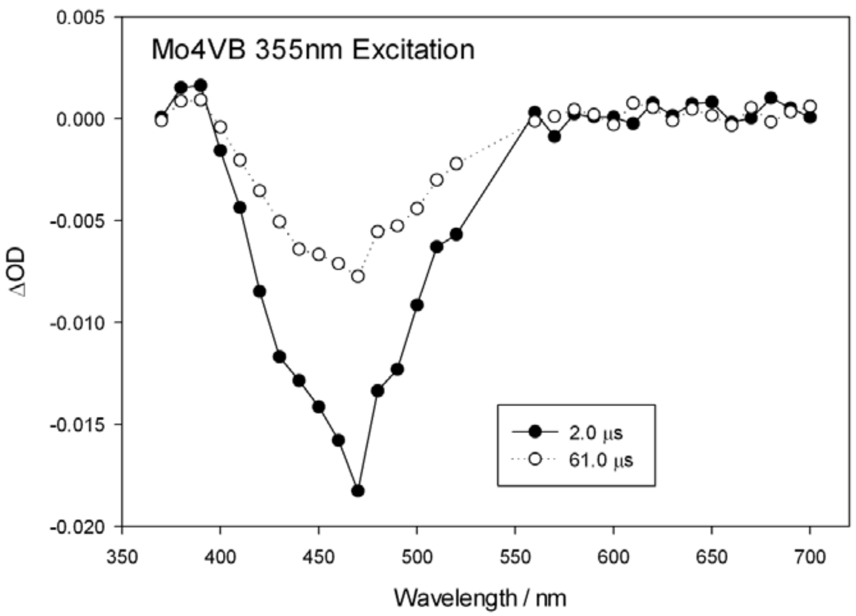

Figure S16. nsTA spectra of **2A** in THF with excitation at 355 nm.

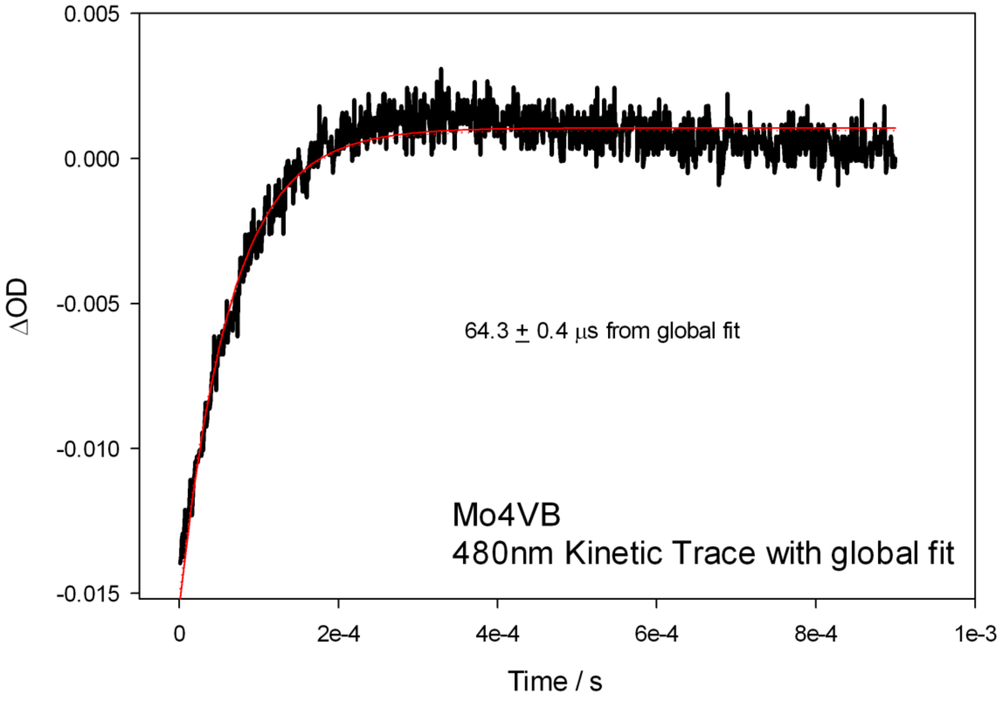

5 Figure S17. Kinetic trace from nsTA spectra of **2A** taken at 480 nm.

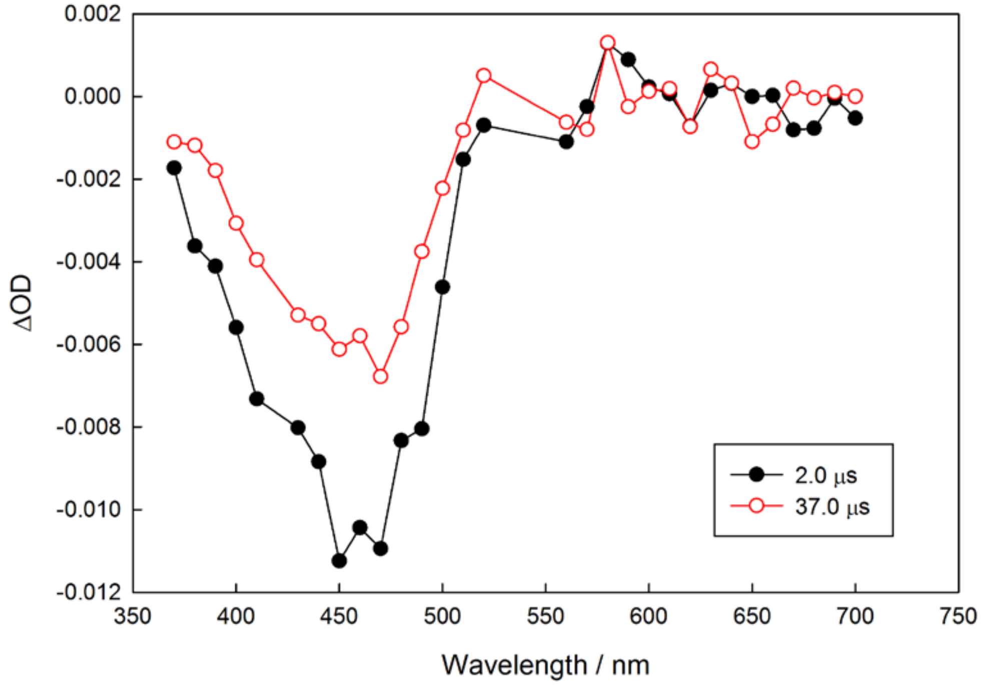

Figure S18. nsTA spectra of **3A** in THF with excitation at 355 nm.

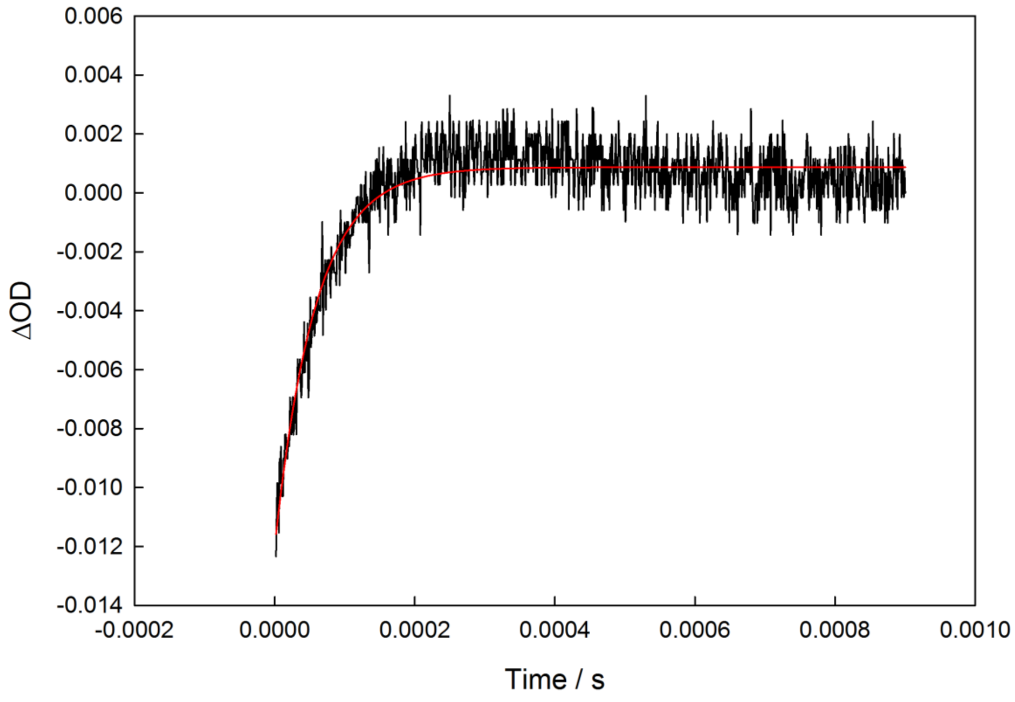

Figure S19. Kinetic trace from nsTA spectra of for **3A** taken at 480 nm.

.

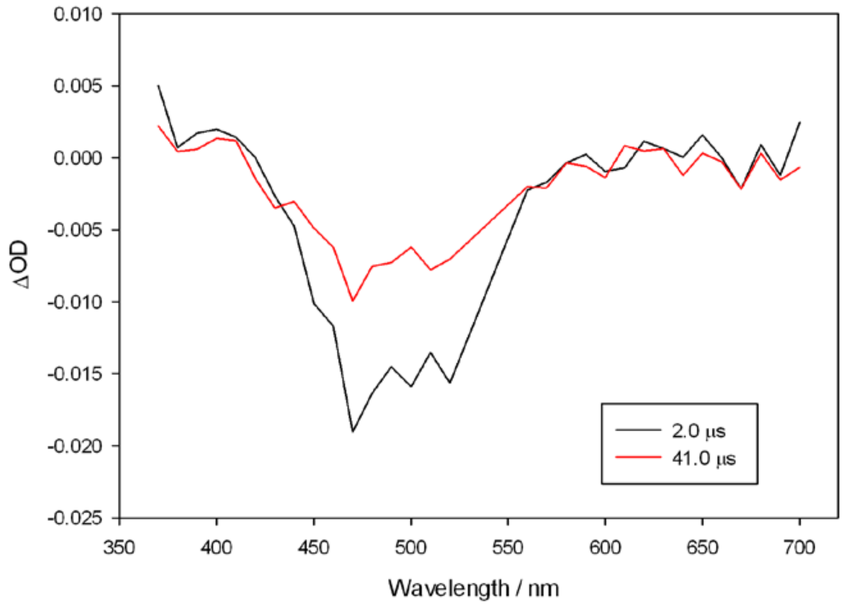

Figure S20. nsTA spectra of **4A** in THF with excitation at 355 nm.

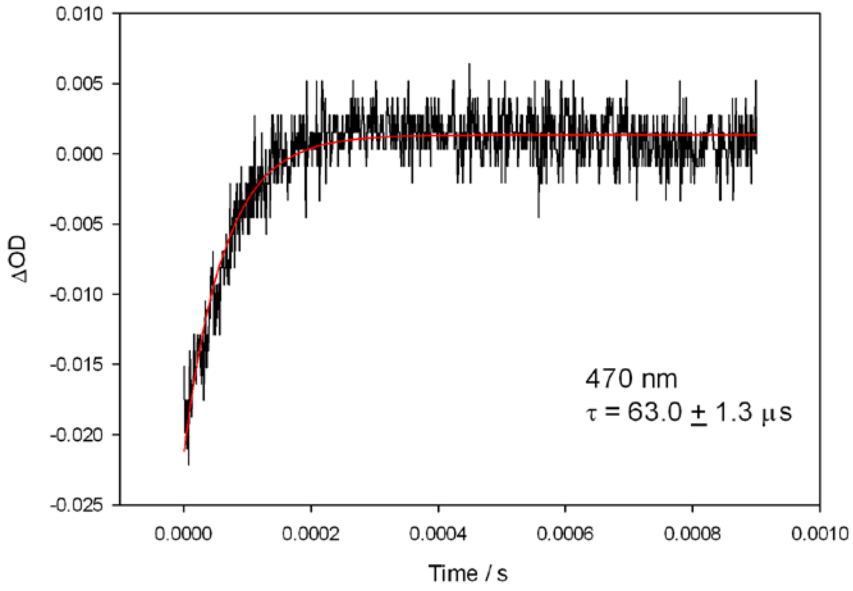

Figure S21. Kinetic trace from nsTA spectra of **4A** taken at 470 nm.

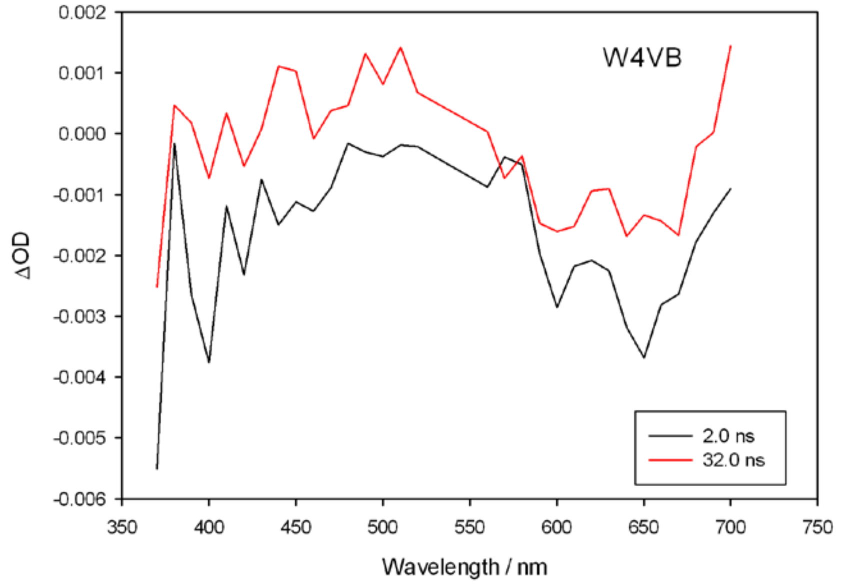

Figure S22. nsTA spectra of **2B** in THF with excitation at 355 nm.

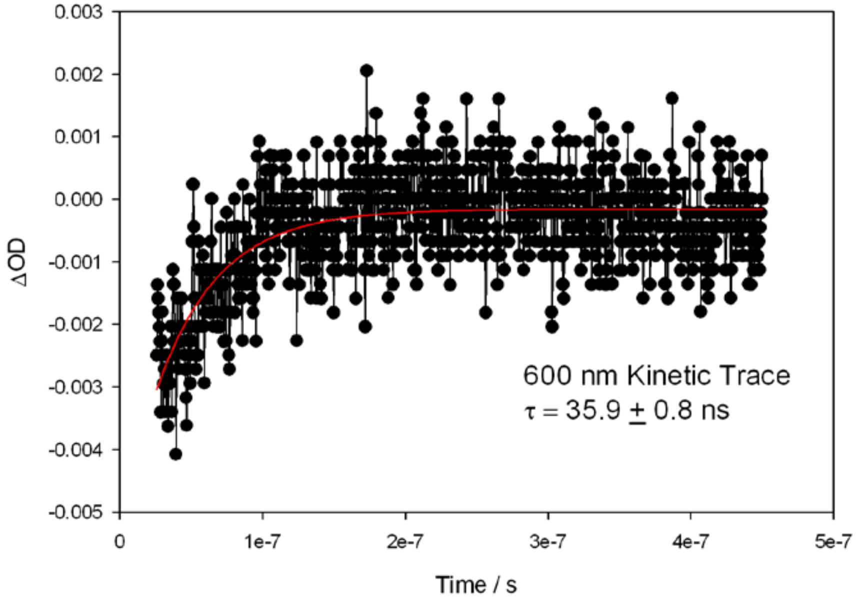

Figure S23. Kinetic trace from nsTA spectra of **2B** taken at 600 nm.

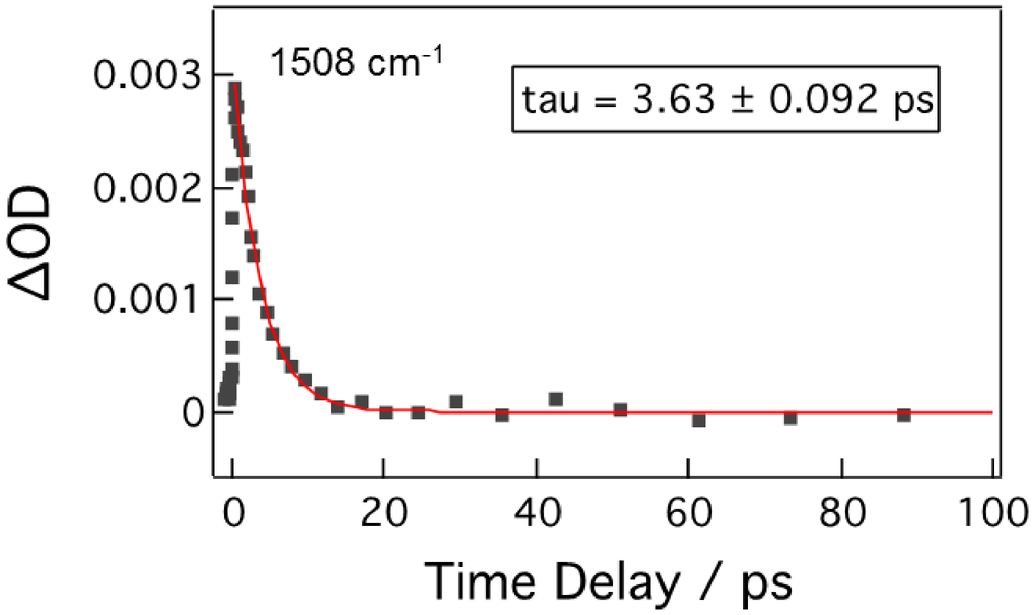

Figure S24. Kinetic trace from fsTRIR spectra of **2A** taken at 1508 cm^{-1}.

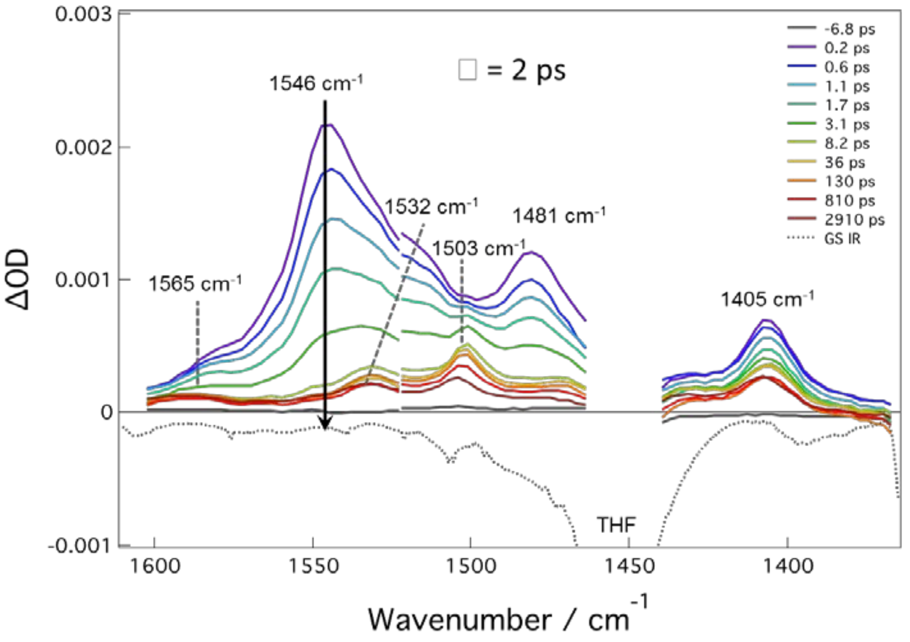

Figure S14. fsTRIR spectra of **2B** in THF with excitation at 675 nm.

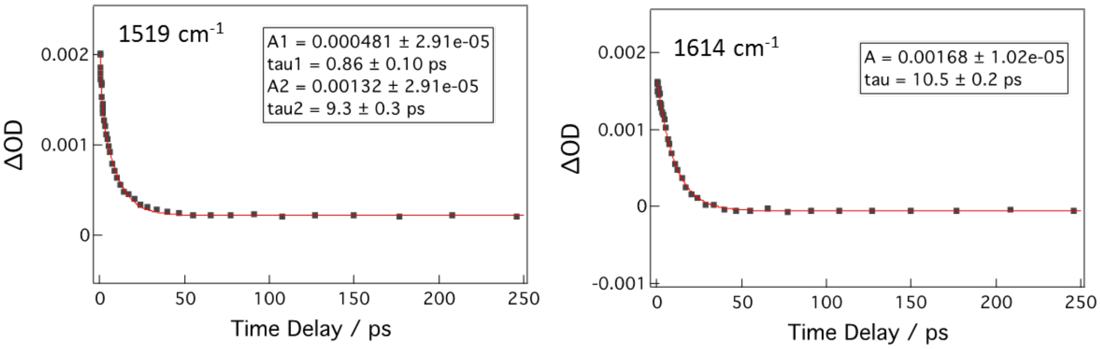

Figure S15. Kinetic traces from fsTRIR spectra of **4A** taken at 1519 and 1614 cm^{-1}.

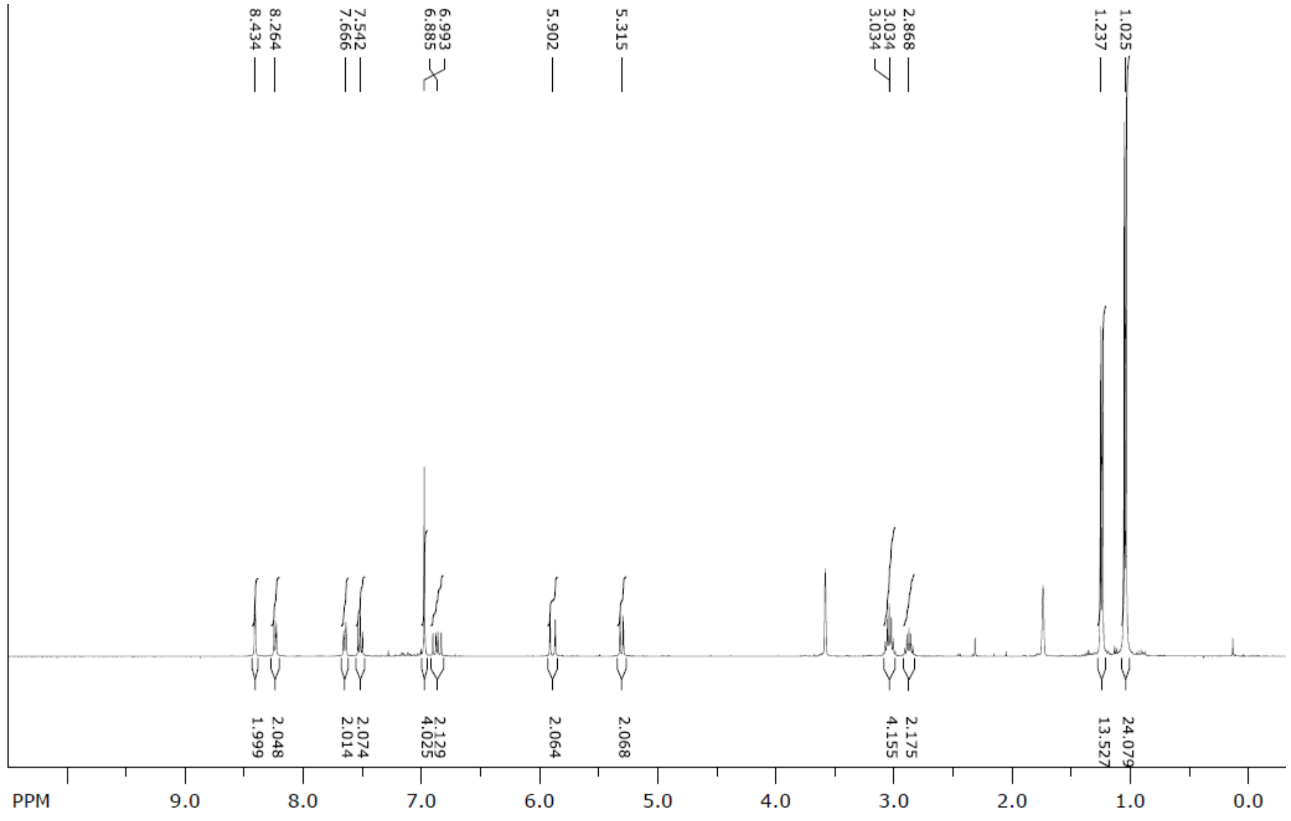

Figure S27. ^{1}H NMR of **1A** in d_8-THF (residual peaks: 3.58 and 1.73 ppm) at room temperature.

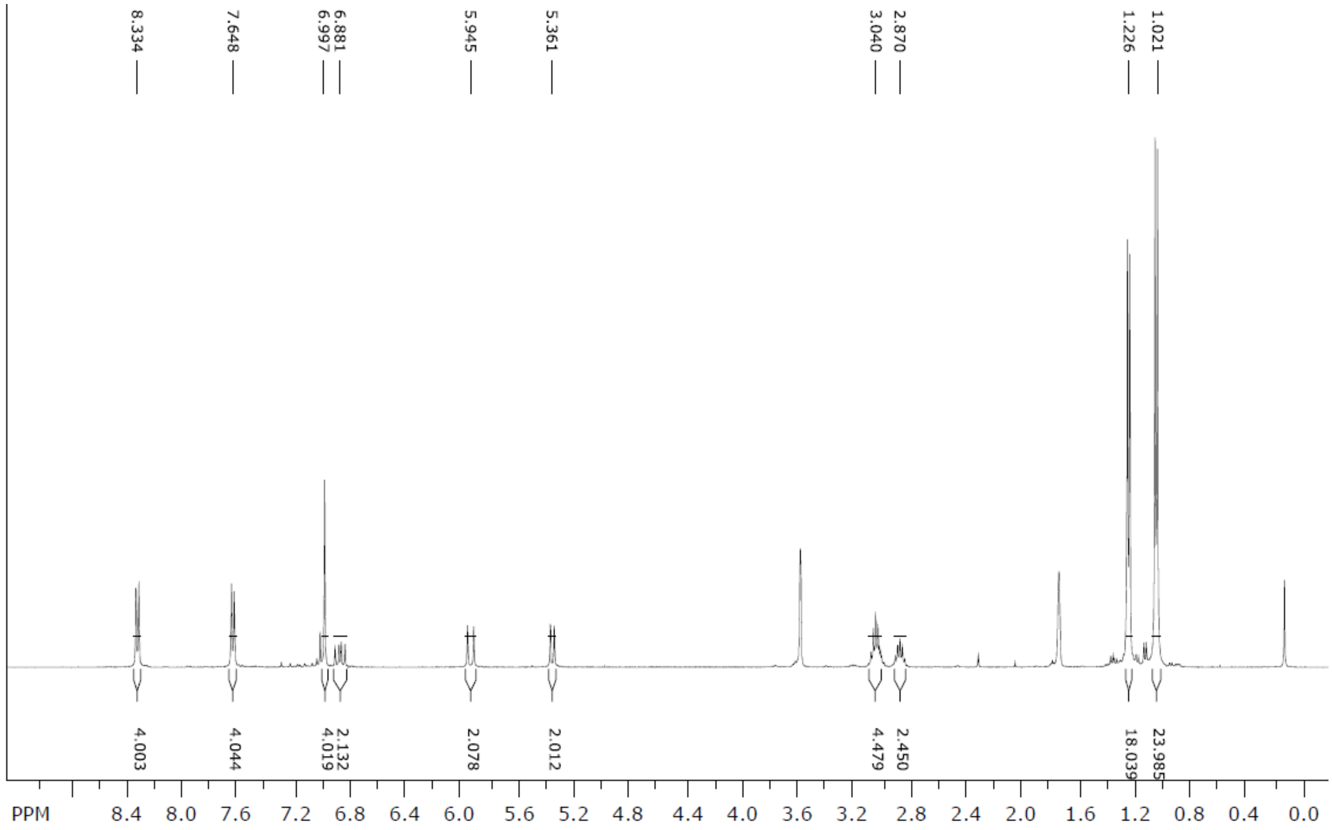

Figure S28. ^{1}H NMR of **2A** in d_8-THF (residual peaks: 3.58 and 1.73 ppm) at room temperature.

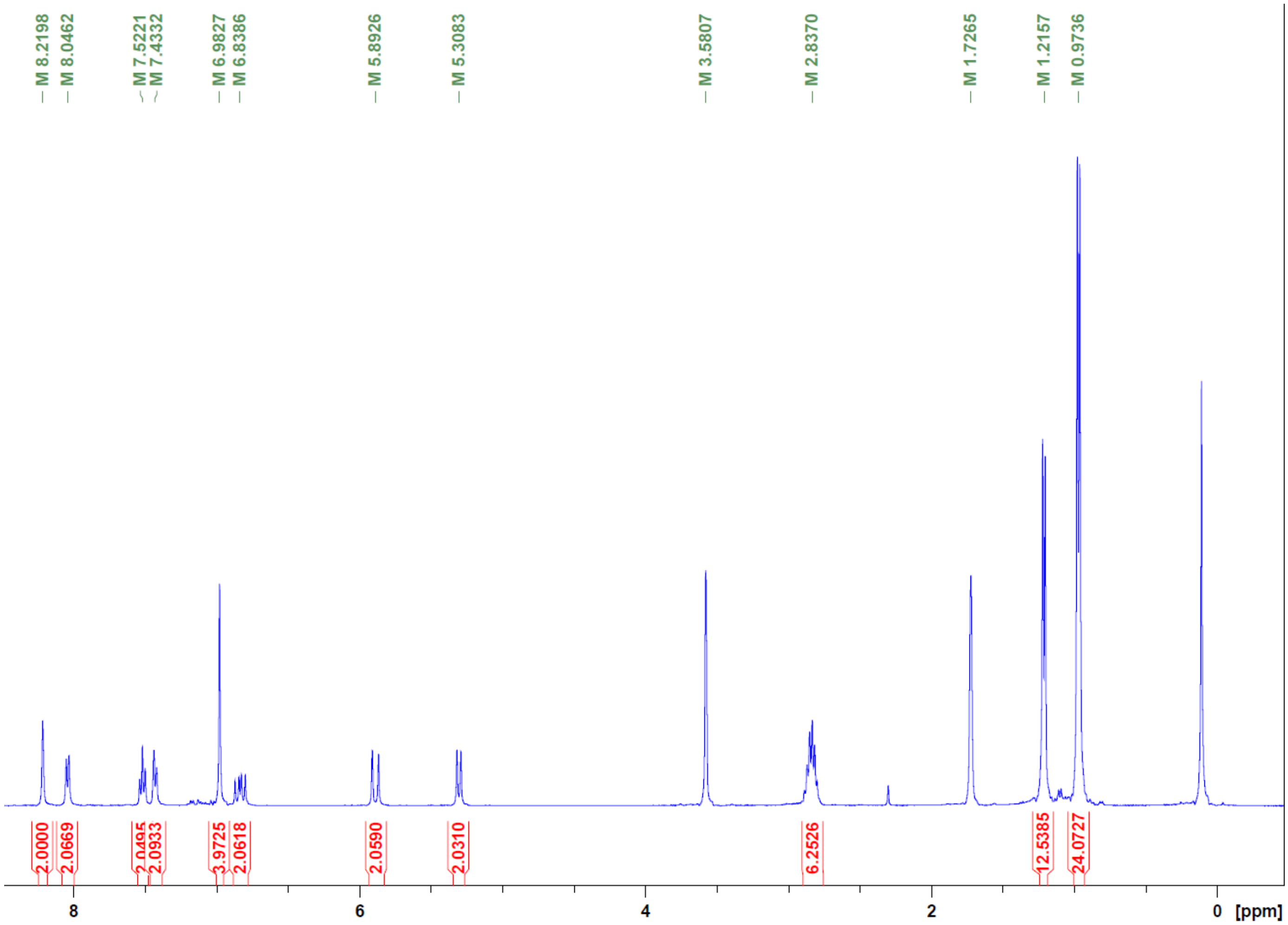

Figure S29 ^{1}H NMR of **1B** in d_8-THF (residual peaks: 3.58 and 1.73 ppm) at room temperature.

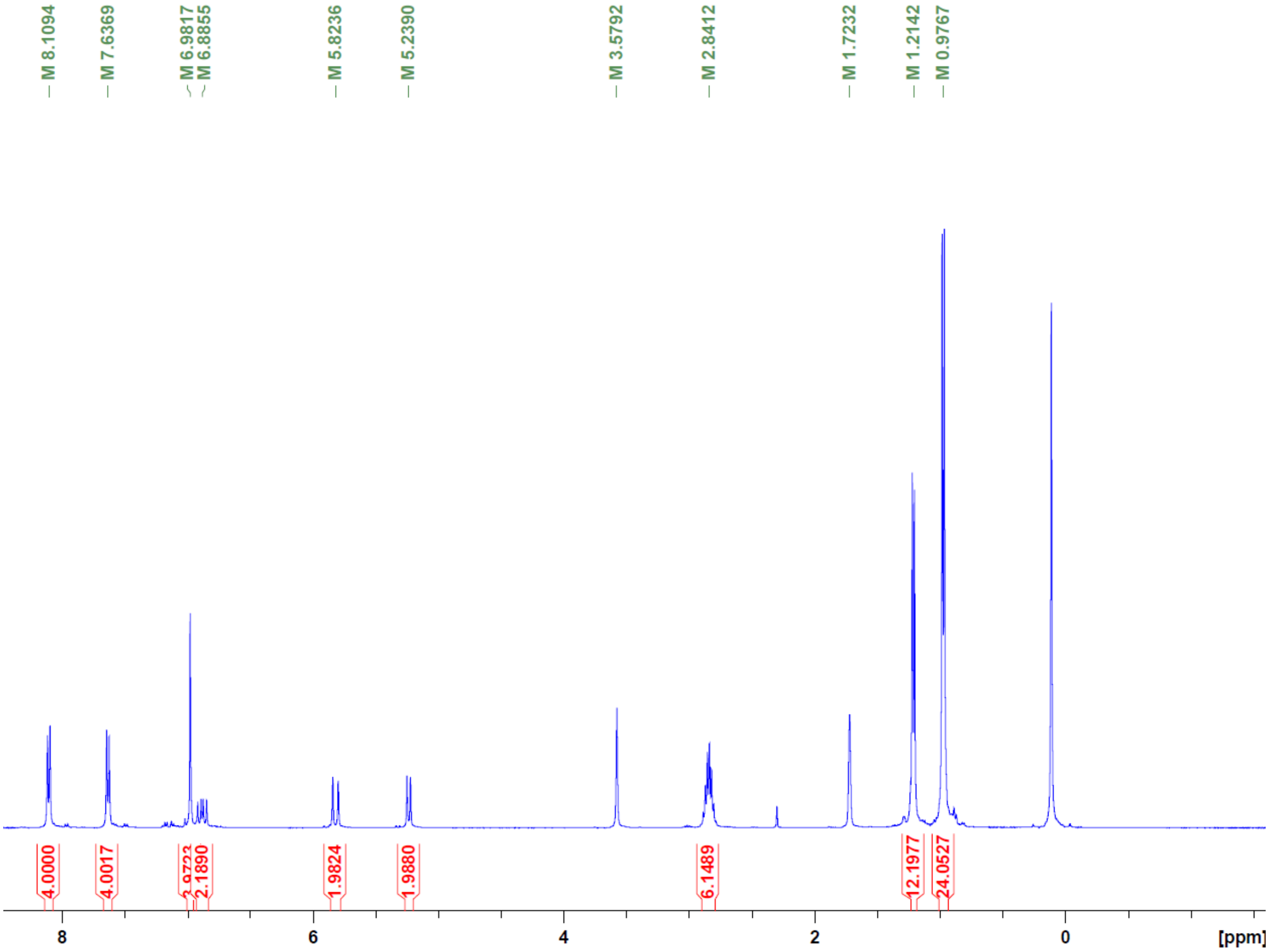

Figure S30. ^{1}H NMR of **2B** in d_8-THF (residual peaks: 3.58 and 1.73 ppm) at room temperature.

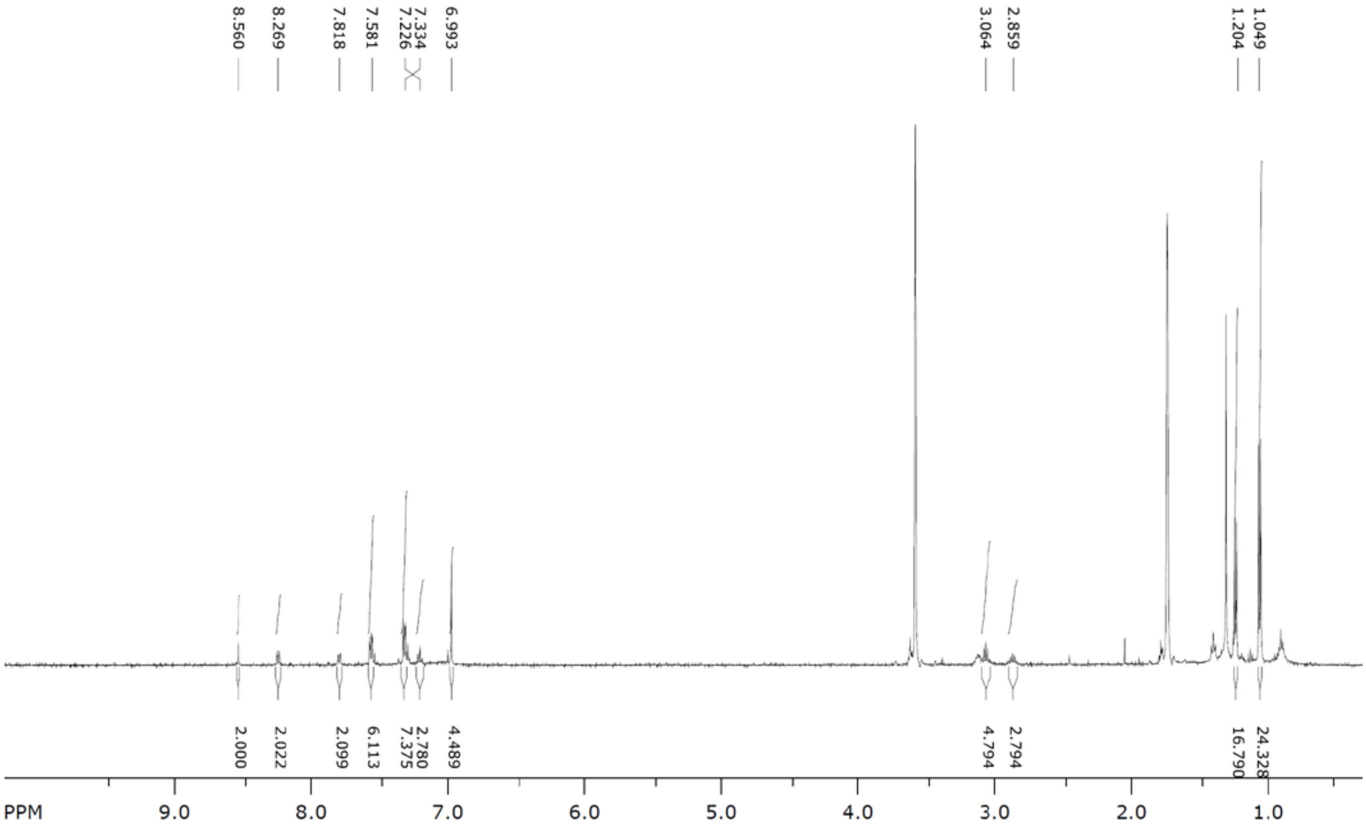

Figure S31. ^{1}H NMR of **3A** in d_8-THF (residual peaks: 3.58 and 1.73 ppm) at room temperature. Small amounts of the catalyst, $Pd(O_2Ac)_2$, can also be seen in the spectra.

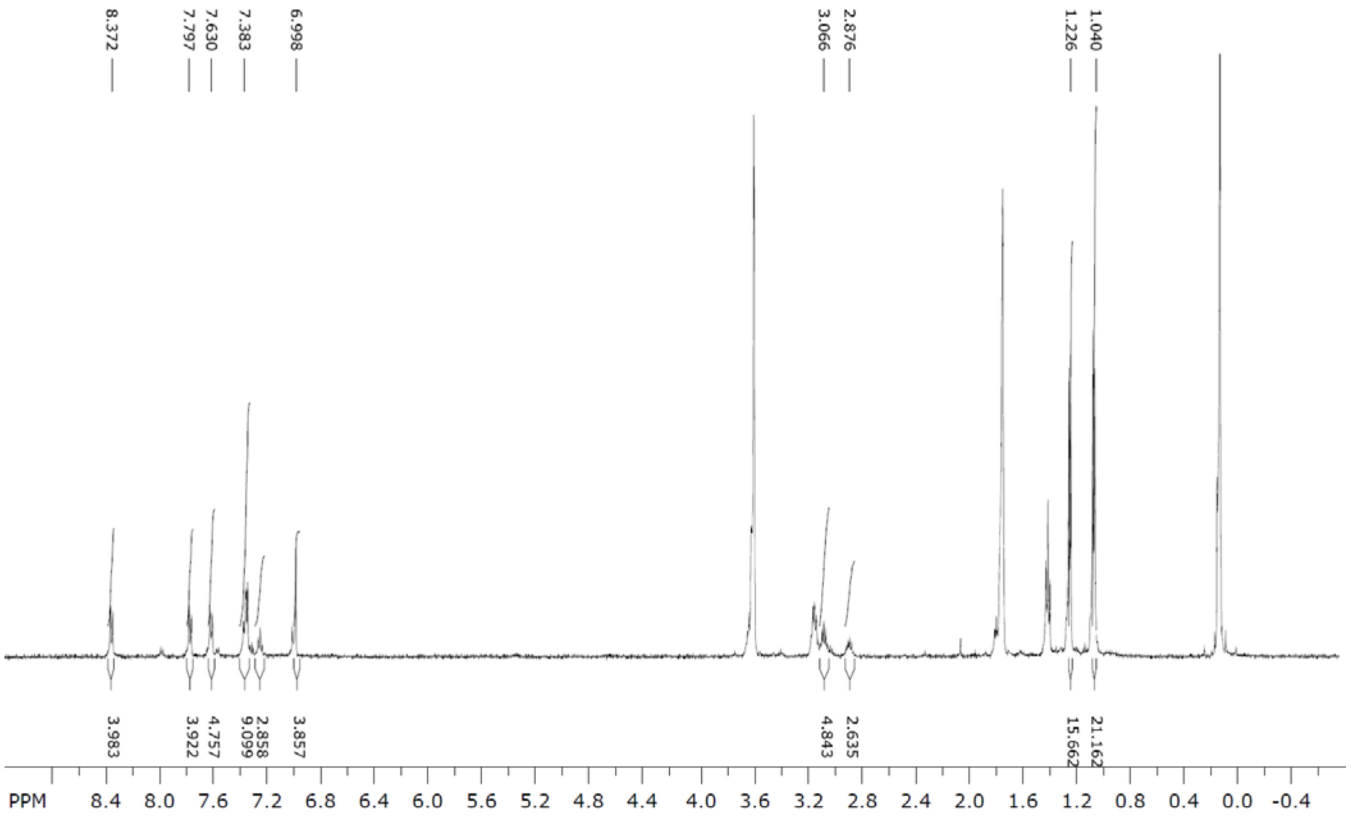

Figure S32. ^{1}H NMR of **4A** in d_8-THF (residual peaks: 3.58 and 1.73 ppm) at room temperature. Small amounts of the base $N(Et)_3$ can also be seen in the spectrum.

Figure S33 High resolution MALDI-MS of **1A**.

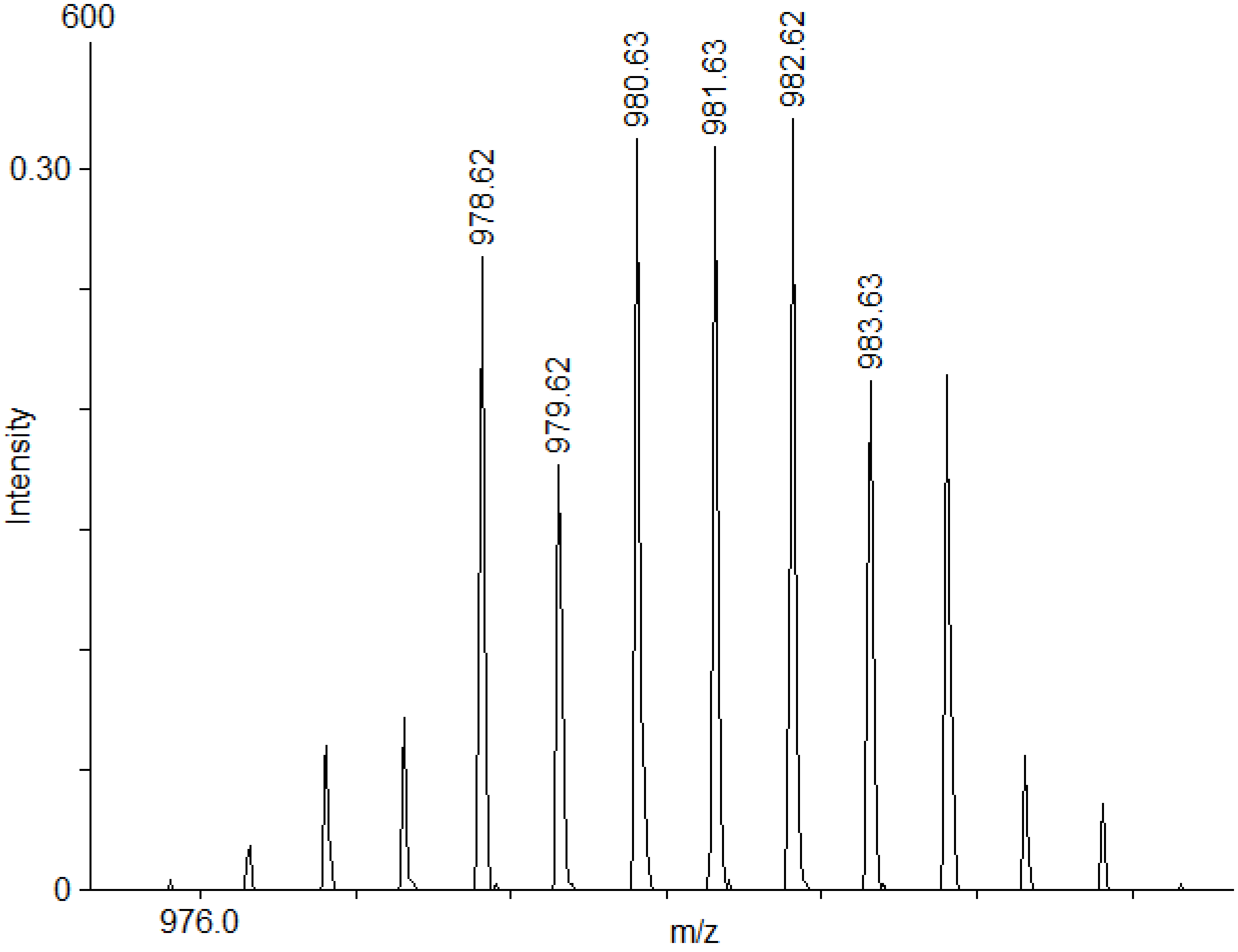

Figure S34 High resolution MALDI-MS of **1B**.

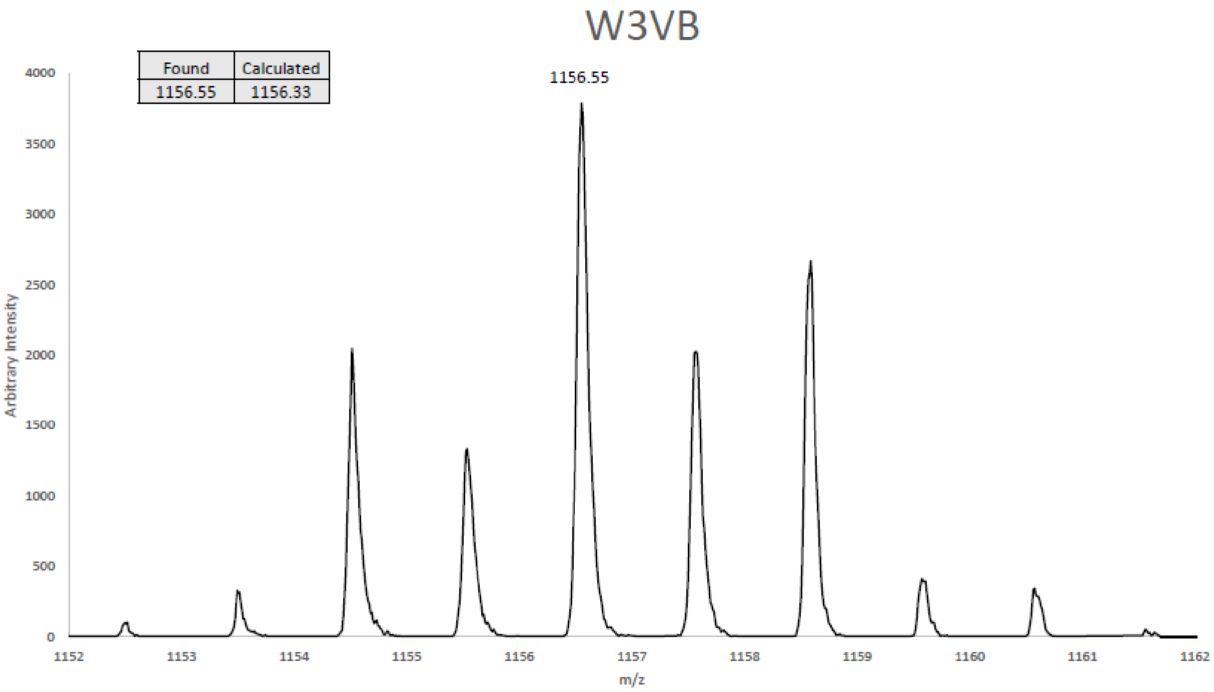

Figure S35 High resolution MALDI-MS of **2A**.

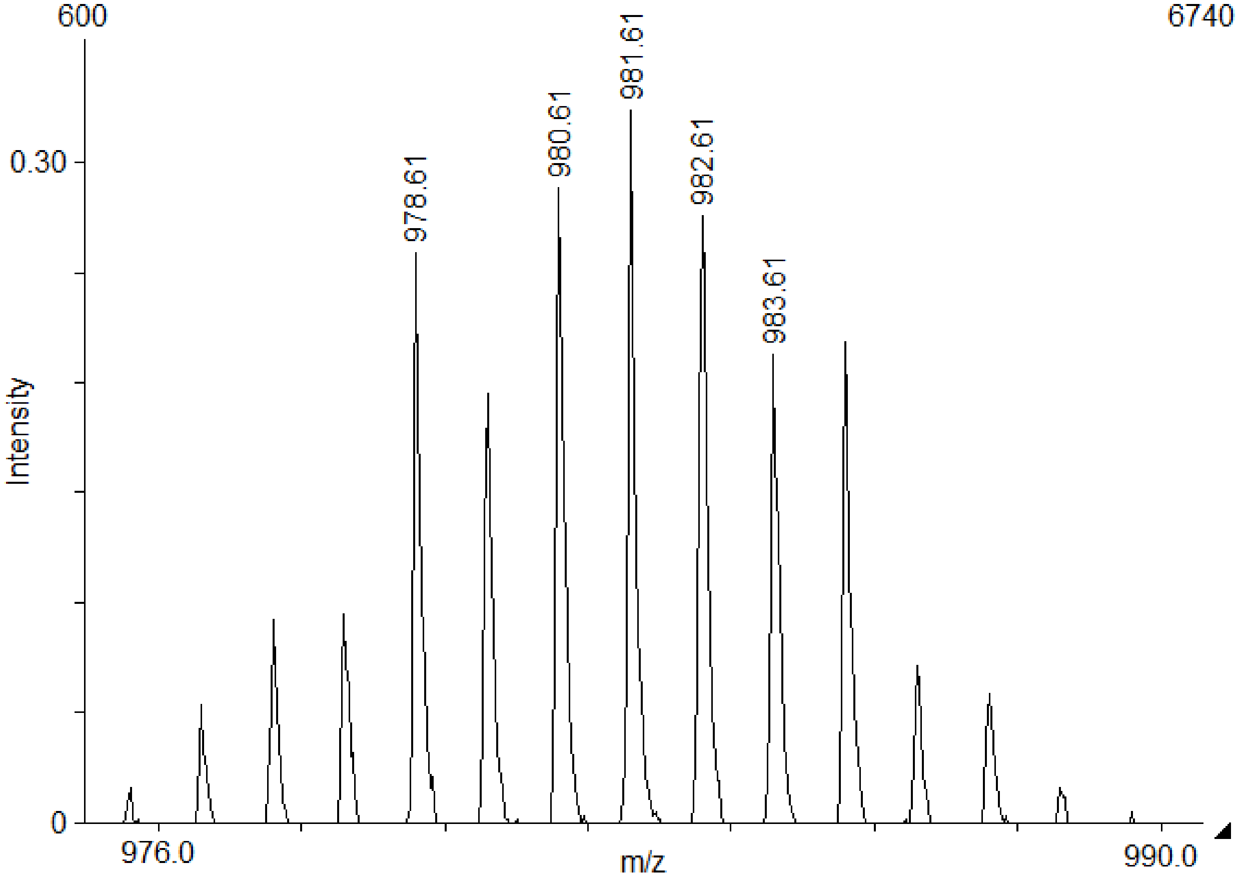

Figure S36 High resolution MALDI-MS of **2B**.

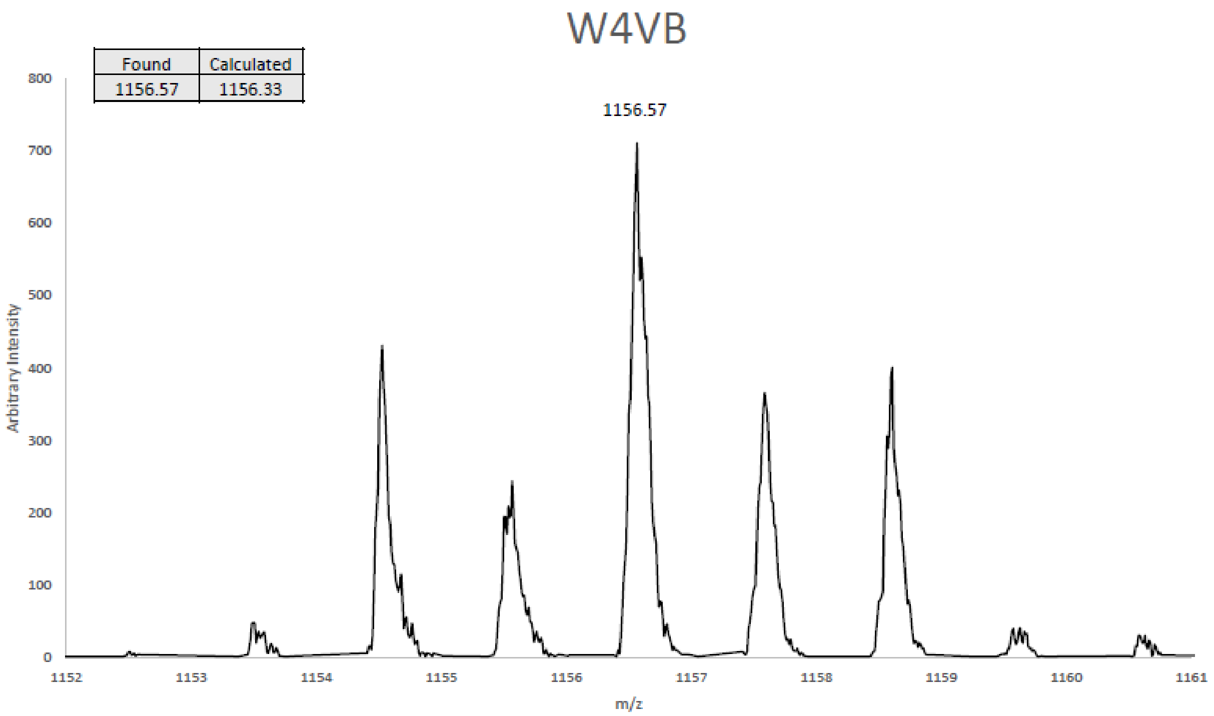

Figure S37 High resolution MALDI-MS of **3A**.

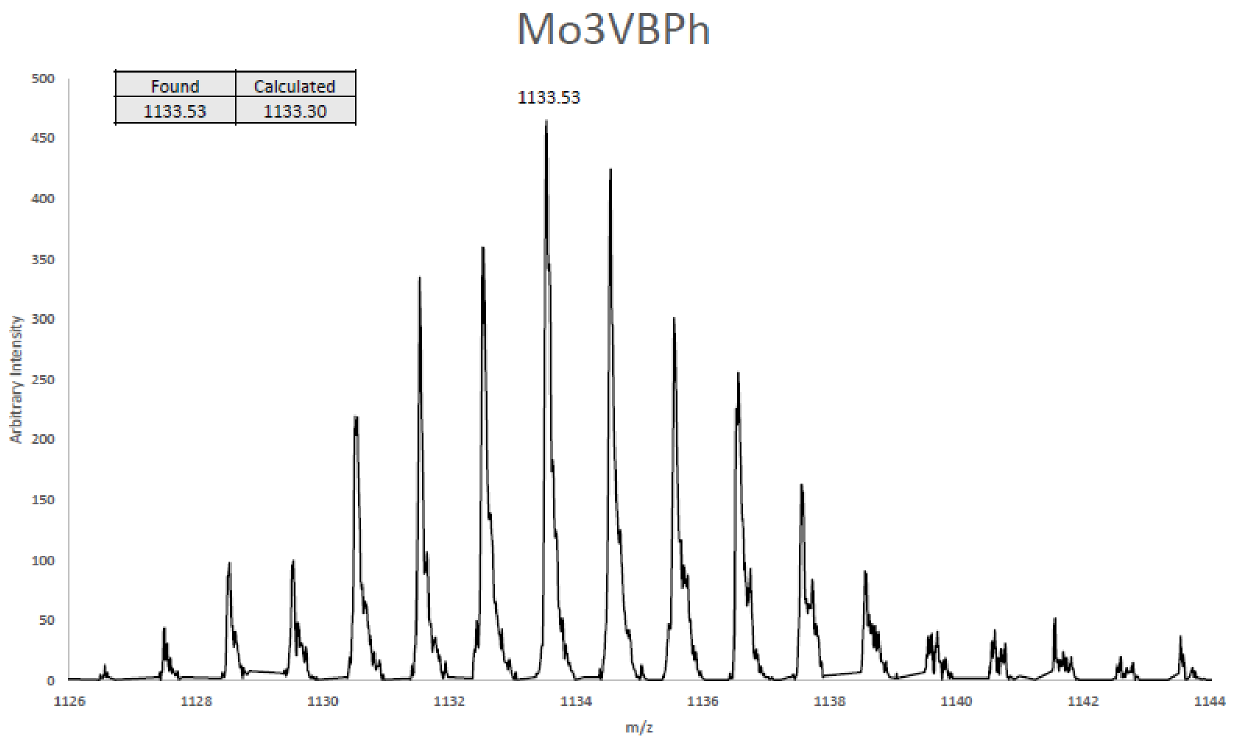

Figure S38 High resolution MALDI-MS of **4A**.

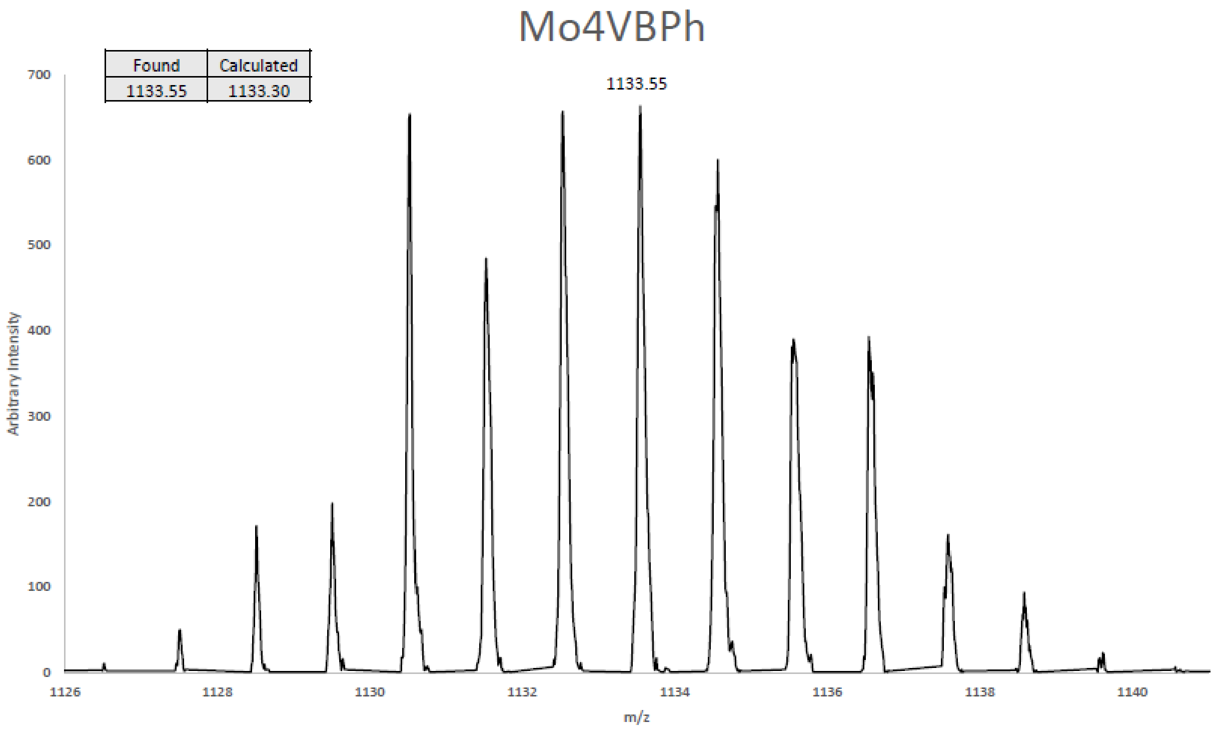

Table S39. Comparison of experimental and theoretical electronic transitions.

Compound	Transition	Experimental Absorption	Calculated Absorption (Oscillator Strength)	Orbital Contribution
**1A**	^{1}MLCT (Mo_2 → T^{i}PB)	337 nm	308 nm (0.1124)	HOMO → LUMO+8
	^{1}MLCT (Mo_2 →3-VB)	437 nm	424 nm (0.5379)	HOMO → LUMO+1
**1B**	^{1}MLCT (W_2 → T^{i}PB)	401 nm	347 nm (0.1526)	HOMO → LUMO+6
	^{1}MLCT (W_2 →3-VB)	563, 601 nm	495 nm (0.6684)	HOMO → LUMO
**2A**	^{1}MLCT (Mo_2 → T^{i}PB)	335 nm	309 nm (0.1099)	HOMO →LUMO+6
	^{1}MLCT (Mo_2 →4-VB)	467 nm	459 nm (0.7307)	HOMO → LUMO
**2B**	^{1}MLCT (W_2 → T^{i}PB)	405 nm	349 nm (0.1488)	HOMO → LUMO+4
	^{1}MLCT (W_2 →4-VB)	605, 652 nm	537 nm (0.8937)	HOMO → LUMO
**3A**	^{1}MLCT (Mo_2 → T^{i}PB)	318 nm	298 nm (0.1136)	HOMO → LUMO+8
	^{1}MLCT (Mo_2 →3-VBPh)	427 nm	373 nm (0.2926)<br>434 nm (0.5829)	HOMO → LUMO<br>HOMO → LUMO+1<br>HOMO → LUMO+3
**4A**	^{1}MLCT (Mo_2 → T^{i}PB)	318 nm	345 nm (1.3354)	HOMO → LUMO+6<br>HOMO-1 → LUMO +1<br>HOMO-2 → LUMO
	^{1}MLCT (Mo_2 →4-VBPh)	486 nm	498 nm (1.2129)	HOMO → LUMO

Table S40. Crystallographic Data Collection Parameters for **1A** and **1B**

Compound	**1A**	**1B**
Chemical Formula	$C_{58}H_{76}Mo_2O_{10}$	$C_{58}H_{76}Mo_2O_{10}$
Formula Weight	1125.06	1125.06
Temperature (K)	150(2)	150(2)
Space Group	Monoclinic, $P2_1/c$	Triclinic, P-1
a (Å)	9.9404(3)	9.8343(2)
b (Å)	15.7440(4)	11.2520(2)
c (Å)	17.7587(4)	15.7140(3)
α (^{o})		107.000(1)
β (^{o})	100.059(1)	105.165(1)
γ (^{o})		90.114(1)
V ($Å^3$)	2736.54 (12)	1599.14(5)
Z	2	1
D_{calcd} (Mg/m^3)	1.365	1.168
Crystal Size (mm)	0.38 X 0.19 X 0.19	0.23 X 0.23 X 0.15
Theta range for data collection	1.740 to 27.433^o	1.899 to 27.467^o
μ (mm^{-1}) [Mo, Kα]	0.514	0.440
F(000)	1176	588
Reflections collected	49649	46246
Unique reflections	6233 [R(int)= 0.051]	7310 [R(int)= 0.038]
Data Completeness to [θ]	100% [25.242]	100% [25.242]
Data/restraints/parameters	6233 / 25 / 343	7310 / 1 / 326
$R1^a$ (%) (all data)	5.06 (7.19)	3.79 (4.96)
$wR2^b$(%)(all data)	11.86 (13.19)	9.78 (10.38)
Goodness-of-fit on F^2	1.058	1.060
Largest diff. peak and hole (e $Å^{-3}$)	1.538 and -0.715	0.559 and -0.477

$^a R1 = \Sigma \,||F_o|-|F_c|| / \Sigma |F_o| \times 100$

$^b wR2 = [\Sigma\, w (F_o^2-F_c^2)^2 / \Sigma (w |F_o|^2)^2]^{1/2} \times 100$
